# Molecular mechanisms by which the intratumoral microbiome drives pancreatic ductal adenocarcinoma progression and therapeutic response

**DOI:** 10.3389/fimmu.2026.1970900

**Published:** 2026-10-22

**Authors:** Zhonghua Qi, Miaolei Wang, Yajing Li, Lina Tian, Junhong Liu

**Affiliations:** 1 Gansu University of Chinese Medicine, Lanzhou, China; 2 Affiliated Hospital of Gansu University of Chinese Medicine, Lanzhou, China

**Keywords:** chemoresistance, immunotherapy, intratumoral microbiome, pancreatic ductal adenocarcinoma, short-chain fatty acids

## Abstract

Pancreatic ductal adenocarcinoma has an extremely poor prognosis. Pancreatic ductal adenocarcinoma harbors a multidomain intratumoral microbial community dominated by bacteria and also containing fungi; its composition differs fundamentally from that of normal pancreatic tissue and drives malignant progression through three major axes: inflammatory-immune signaling, metabolic-epigenetic regulation, and stromal remodeling. Gammaproteobacterial lipopolysaccharide establishes a chronic proinflammatory microenvironment through the Toll-like receptor 4-nuclear factor kappa B axis, whereas *in situ* gemcitabine inactivation by the long isoform of bacterial cytidine deaminase represents the best-supported microbiome-mediated mechanism of drug resistance to date. In experimental systems, Fusobacterium nucleatum has been linked to tumor-cell invasion and alternatively activated macrophage polarization. In mouse and cell models, intratumoral fungi have been implicated in immunosuppressive signaling through the C-type lectin receptor dectin-1-interleukin 33 axis and the mannose-binding lectin-complement pathway; their relevance to human pancreatic ductal adenocarcinoma remains debated. These three axes may converge on stromal activation and epithelial-mesenchymal transition and contribute to invasion and treatment resistance. Antimicrobial strategies targeting drug-resistance-associated bacteria can restore gemcitabine efficacy in animal experiments and have been explored experimentally alongside programmed cell death protein 1 blockade; the clinically appropriate patient populations and optimal sequencing of such combinations remain to be defined in prospective trials.

## 1 Introduction

Pancreatic ductal adenocarcinoma (PDAC) accounts for approximately 85%-90% of pancreatic malignancies and is the seventh leading cause of cancer-related death worldwide (1, 2). Over the past decade, the 5-year overall survival rate of PDAC has increased only slowly, from approximately 6% to approximately 12%, reflecting extremely limited therapeutic progress compared with other major malignancies. More than 80% of patients have locally advanced or metastatic disease at diagnosis, and fewer than 20% are eligible for surgical resection. The dense desmoplastic stroma generated predominantly by activated pancreatic stellate cells not only impedes the penetration of chemotherapeutic agents but also actively excludes effector T cells from tumors through the C-X-C motif chemokine ligand 12-C-X-C motif chemokine receptor 4 axis (3). A multilayered immunosuppressive network dominated by myeloid-derived suppressor cells, alternatively activated (M2-like) tumor-associated macrophages, and regulatory T cells contributes to objective response rates below 5% for immune-checkpoint inhibitors (4, 5). These characteristics make PDAC one of the most treatment-refractory solid tumors and underscore the need to identify new determinants of pathogenesis and treatment resistance.

Systematic investigation of the relationship between microorganisms and cancer has advanced markedly in recent years. Using rigorous decontamination procedures, Nejman et al. systematically analyzed 1,526 tumors and matched normal tissues across seven cancer types and demonstrated distinct intracellular microbial communities, localized mainly within cancer and immune cells, thereby establishing the intratumoral microbiome as a distinct biological entity (6). This cancer-type specificity was further confirmed by subsequent pan-cancer studies and systematic reviews of different solid tumors: each cancer type exhibits a relatively characteristic microbial composition, and the diversity and composition of intratumoral microbial communities are associated with tumor stage, immune-cell infiltration, and patient prognosis (7, 8). Considering both bacterial and fungal dimensions, the tumor microbiome is now regarded as an independent functional component of the tumor microenvironment rather than merely opportunistic contamination (8). Unlike many other gastrointestinal organs, the healthy pancreas is not traditionally regarded as a microbial habitat. Geller et al. detected bacterial deoxyribonucleic acid (DNA) in approximately 76% of PDAC tissues but in only approximately 15% of normal pancreatic tissues, suggesting selective expansion of specific microorganisms during tumor development (9). Subsequent independent studies have confirmed that PDAC contains multidomain intratumoral communities dominated by bacteria and also containing fungi, which participate in tumorigenesis, immune escape, and chemoresistance through multiple mechanisms (10–12). Existing reviews of the PDAC intratumoral microbiome have often organized the literature by broad mechanistic category, making it difficult to follow the complete regulatory pathway of individual microorganisms. At the same time, the field faces challenges including contamination in low-biomass samples, limitations of causal inference in cross-sectional studies, and population-dependent microbial heterogeneity (13, 14). Mendelian-randomization studies have provided new evidence regarding causal links between the gut microbiota and PDAC (15). This review therefore uses molecular pathways as its organizing framework and progressively examines the specific mechanisms and regulatory consequences of different microorganisms across inflammatory-immune signaling, metabolic-epigenetic regulation, and stromal remodeling-epithelial-mesenchymal transition (EMT), while integrating relevant translational therapeutic strategies.

## 2 Composition of the intratumoral microbiome in PDAC

The PDAC intratumoral microbial ecosystem comprises bacteria, fungi, and other microorganisms colonizing pancreatic tumor tissue and is distinct from the intestinal microbiome or circulating microbial DNA. These microorganisms can reside within tumor cells, immune cells, and stromal compartments, and their composition differs fundamentally from that of normal pancreatic tissue. At the phylum level, the bacterial compartment is dominated by Proteobacteria, whereas Malassezia and Candida are among the fungi commonly detected; compositional and functional interactions may also occur between bacteria and fungi (16, 17). This ecosystem may be established through multiple colonization routes originating from the gut and oral cavity and exhibits marked spatial heterogeneity across tumor regions. Because the total microbial biomass within tumors is extremely low, sequencing data based on relative abundance may not reflect the true microbial load; targeted absolute-quantification amplicon sequencing provides a new means of comparing community abundance and microbial load across studies (18). Estimates of intratumoral microbial load and composition vary substantially among studies, closely related to differences in sample type, sequencing target region, DNA-extraction method, and decontamination strategy (19, 20). Accordingly, the compositional features described here represent overall trends in the current literature rather than quantitatively comparable conclusions across studies. Defining community composition, colonization routes, and spatial distribution is a prerequisite for understanding the functional regulation exerted by intratumoral microorganisms. The proposed routes of microbial entry and the spatial organization of the intratumoral microbiome are illustrated in **Figure 1**.

**Figure 1:**
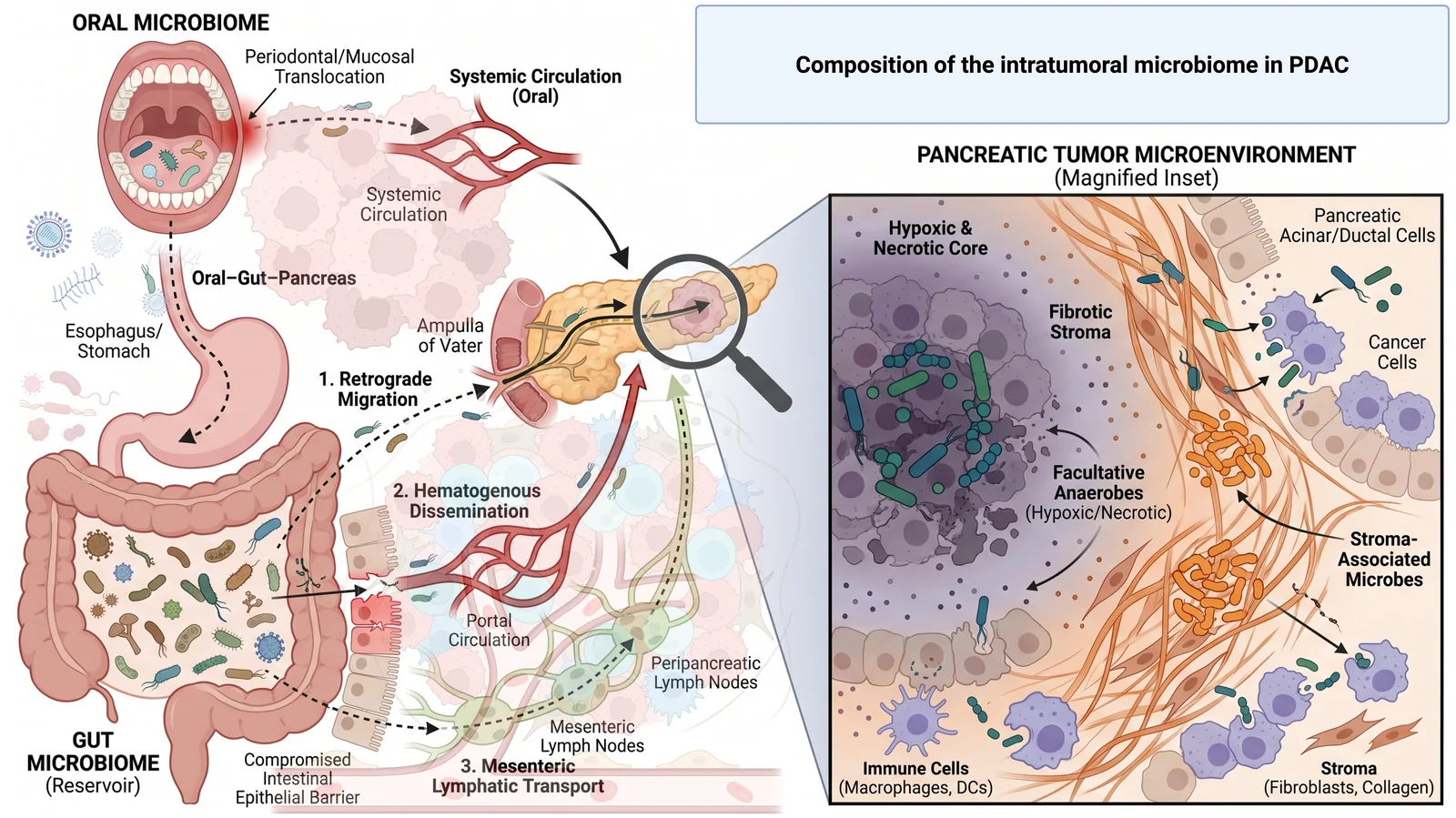
Proposed routes of microbial colonization and spatial organization of the intratumoral microbiome in PDAC. Microorganisms detected in PDAC may originate from several non-mutually exclusive sources. Gut microorganisms can potentially reach the pancreas through retrograde migration along the duodenum-pancreatic duct axis, translocation across a compromised intestinal barrier followed by hematogenous dissemination, or mesenteric lymphatic transport. Oral microorganisms may reach pancreatic tissue through the oral-gut-pancreas route or systemic circulation. Within the tumor, microorganisms can localize to cancer cells, immune cells, and stromal compartments and may exhibit spatial enrichment according to local oxygen tension, necrosis, fibrosis, and nutrient availability. Hypoxic or necrotic regions may favor facultative or anaerobic organisms, whereas distinct stromal niches may support different microbial communities. The proposed routes differ substantially in the strength of the supporting evidence. Retrograde migration from the duodenum into the pancreatic parenchyma and hematogenous translocation of gut-derived bacteria have been directly demonstrated in mouse models, whereas the oral-gut-pancreas axis, mesenteric lymphatic transport, and systemic dissemination of oral microorganisms are supported mainly by indirect epidemiological, serological and sequencing evidence and have not yet been directly validated in PDAC-specific models. These routes are therefore presented as proposed or partially supported rather than as established mechanisms, and the model system on which each route is based is specified in the corresponding section of the main text.

### 2.1 Microbial composition and abundance

At the phylum and class levels, the PDAC intratumoral microbial ecosystem is characterized by a predominance of Proteobacteria, particularly Gammaproteobacteria such as Enterobacteriaceae, Klebsiella, and Pseudomonas, together with Alphaproteobacteria, followed by Firmicutes and Bacteroidetes (21). Compared with normal pancreatic tissue, overall alpha diversity within PDAC tumors is significantly reduced, reflecting selective enrichment of specific tumor-promoting microorganisms rather than a generalized increase in microbial diversity. Riquelme et al. systematically compared intratumoral microbial communities in long-term survivors (median survival >5 years) and short-term survivors and found significantly greater intratumoral alpha diversity in the long-term-survivor group, with selective enrichment of Saccharopolyspora, Pseudoxanthomonas, Streptomyces, and Bacillus clausii. The abundance of these taxa was positively correlated with cluster of differentiation 8 (CD8)-positive T-cell infiltration and interferon-gamma production in the tumor microenvironment (22). At the species level, Fusobacterium nucleatum, one of the most extensively studied tumor-promoting bacteria in gastrointestinal malignancies, has been detected in approximately 8.8% of PDAC tissues, with rates of 15%-20% reported in some pan-cancer analyses, and its presence is associated with shorter overall survival (23). In the fungal compartment, Aykut et al. reported an approximately 3,000-fold increase in fungal abundance in resected PDAC tissue and Kras/Trp53/Pdx1-Cre transgenic mouse tumors compared with normal pancreas, with Malassezia globosa as a dominant species and a composition partly independent of the bacterial community (17). In human specimens, recent studies have directly isolated and cultured bacteria and fungi from resected PDAC specimens and matched duodenal fluid, providing culture-based evidence for the actual presence of intratumoral microorganisms, although the number and diversity of cultivable microorganisms are markedly lower than the community size inferred by sequencing (24). Jayakrishnan et al. found that higher bacterial diversity in tumors from patients with early-onset PDAC (age <50 years) was associated with improved overall survival (25). PDAC can also be classified into distinct microbial subtypes based on community composition, with significant differences in immune-microenvironment characteristics and clinical outcomes (26). Malassezia is not the only fungal member detected in PDAC. Candida is a common fungus of the intestinal and biliary systems and is significantly enriched in the fecal mycobiome of patients with chronic pancreatitis (27). However, in fungal recolonization experiments by Aykut et al., only Malassezia accelerated pancreatic tumor growth in mice, whereas recolonization with Candida, Saccharomyces, or Aspergillus had no such effect, indicating pronounced species specificity in fungal tumor-promoting activity (17). The reproducibility of fungal signals in human PDAC has also been questioned: an independent reanalysis of the public sequencing data from Aykut et al. failed to confirm differences in fungal communities between normal pancreatic and PDAC tissues and detected very few fungal sequences (28). The original investigators responded by emphasizing differences in bioinformatic workflows and functional validation (29), and discrepancies in fungal detection across studies can be partly attributed to sequencing and decontamination strategies under low-biomass conditions. In addition, recent work has resolved intratumoral bacterial chemoresistance to a specific species: Enterobacter hormaechei isolated from PDAC tissue can mediate gemcitabine inactivation through the long isoform of bacterial cytidine deaminase (CDD_L) (30). The major intratumoral microorganisms reported in PDAC, their detection patterns, and their clinical associations are summarized in **Table 1**.

**Table 1:** Major intratumoral microorganisms reported in PDAC, their detection patterns, and clinical associations.

Microorganism	Domain	Detection/change in PDAC	Clinical association
Gammaproteobacteria (Enterobacteriaceae, Klebsiella, Pseudomonas)	Bacteria	Dominant intratumoral community in PDAC; significantly increased abundance	Associated with gemcitabine resistance; CDD_L mediates drug inactivation
Fusobacterium nucleatum	Bacteria	Detected in approximately 8.8%; 15%-20% in some studies	Associated with shorter overall survival; promotes proliferation and migration
Bacteroides spp.	Bacteria	Anaerobic-community component; selected strains enriched	Associated with reduced T-cell infiltration and poorer prognosis
Peptoniphilus spp.	Bacteria	Detected in a subset of PDAC tumors	Independent prognostic factor (hazard ratio 3.48); associated with T-cell suppression
Malassezia globosa	Fungi	Reported enrichment in one sequencing study; low-biomass human signals remain disputed	Mouse/cell-model effects via mannose-binding lectin-complement and interleukin 33 pathways; human causal relevance unconfirmed
Saccharopolyspora/Pseudoxanthomonas/Streptomyces/Bacillus clausii	Bacteria	Enriched in long-term survivors	Associated with favorable prognosis and greater CD8-positive T-cell infiltration
Blautia	Bacteria	Enriched in some PDAC tissues	Associated with favorable prognosis; 6-hydroxyhexanoic acid promotes classically activated polarization
Candida spp.	Fungi	Common fungi of the intestinal and biliary systems; enriched in the fecal mycobiome of chronic pancreatitis; low intratumoral abundance in PDAC with inconsistent findings among studies	Mouse recolonization experiments did not show tumor-promoting effects; tumor-promoting activity is strongly species specific
Enterobacter hormaechei	Bacteria	A Gammaproteobacterial species that can be isolated and cultured from PDAC tissue	Mediates gemcitabine inactivation through the CDD_L; associated with chemoresistance

The included populations, sample types, sequencing methods, and decontamination strategies differ among studies; therefore, detection frequencies and changes in abundance shown in the table should not be directly compared across studies. The reproducibility of fungal findings in human specimens remains controversial. In the clinical-association column, associations between bacterial abundance and prognosis or drug resistance are derived from human PDAC cohorts and surgical specimens, whereas the functional conclusions regarding Malassezia and Candida are derived from mouse models and cell lines.

### 2.2 Potential routes of colonization

Intratumoral microorganisms may originate through several non-mutually exclusive colonization routes. The principal proposed route is retrograde dissemination along the duodenum-pancreatic duct axis. In Kras/Trp53/Pdx1-Cre mice, Pushalkar et al. used orally administered fluorescently labeled bacteria to demonstrate that intestinal bacteria could migrate retrogradely from the duodenum into the pancreatic parenchyma within hours (31). Direct evidence for this retrograde route currently comes mainly from fluorescence-labeling or tracing experiments in mouse models, whereas its frequency and temporal characteristics in human PDAC remain unclear (32). A second route involves hematogenous dissemination after translocation across a compromised intestinal mucosa and transport through mesenteric lymphatics (33). Using whole-genome sequencing, Zhao et al. recently provided direct evidence of bacterial translocation from the gut to tumor tissue: multidrug-resistant Klebsiella pneumoniae could migrate from the intestine to PDAC tissue and pancreatic juice while carrying antibiotic-resistance genes including acrB, mdtC, and cpxA (34). In experimental mice, intestinal bacteria have been shown to translocate to multiple internal organs traditionally considered sterile, including the pancreas, providing experimental support for hematogenous and lymphatic routes (32). A third route is the oral-gut-pancreas axis. Oral pathogens may enter the intestine by swallowing and subsequently reach the pancreas through the routes above, or may directly enter the bloodstream through periodontal microvasculature and disseminate hematogenously to the pancreas. In a large European prospective cohort, Michaud et al. found that elevated serum immunoglobulin G antibodies against Porphyromonas gingivalis were associated with increased pancreatic-cancer risk (35). Prospective cohorts support associations between oral microbiota and pancreatic cancer (36, 37), and significant alterations have also been reported in duodenal and fecal communities (38, 39), increasing interest in the translational relevance of the oral-gut microbial axis in PDAC (40). The association between intestinal dysbiosis and pancreatic cancer involves interactions among immune, metabolic, and microbial signals, providing an integrated perspective on the gut-pancreas axis (41). Spatial-omics approaches can further resolve microbe-host interactions (42, 43). Gaiser et al. detected significant enrichment of oral-derived bacteria in early cystic precursor lesions of PDAC, namely intraductal papillary mucinous neoplasms, suggesting that microbial colonization may precede overt PDAC (44). Spatial microbiome analyses indicate that different regions of a single PDAC tumor can harbor distinct communities at both phylum and genus levels. Necrotic or hypoxic regions tend to be enriched in facultative anaerobes such as Klebsiella and Pseudomonas, whereas fibrotic stromal regions are dominated by microorganisms tolerant of oxidative stress (42). This spatial heterogeneity suggests that microbiome analysis based on a single-site needle biopsy may underestimate overall intratumoral microbial diversity. Plasma microbial cell-free DNA has also shown preliminary potential as a noninvasive liquid-biopsy signal for tumor detection and classification (7).

A substantial proportion of mechanistic evidence in this field derives from studies of the gut microbiome; however, the PDAC intratumoral microbiome differs substantially from the intestinal microbiome in community composition, metabolite concentrations, local immune environment, and oxygen tension, and this biological distance should not be overlooked. Some studies infer changes in the intratumoral microenvironment from alterations in the gut microbiota, an inference that assumes that microorganisms or their metabolites can effectively reach tumor tissue through retrograde migration along the duodenum-pancreatic duct axis, hematogenous spread across a compromised intestinal barrier, or lymphatic transport. In human PDAC, the extent to which this prerequisite is fulfilled is supported mainly by tracing experiments in mouse models. For short-chain fatty acids, secondary bile acids, and their receptors, the relevant functional evidence derives mainly from colonic epithelium, intestinal immune cells, or extraintestinal tumor models; the local concentrations achieved within PDAC tumors and the specific responding cell types remain uncertain, and these findings therefore cannot simply be extrapolated as direct effects of intratumoral microorganisms (45–47). Differences among clinical cohorts likewise require cautious interpretation: host genetic background, dietary structure, sample processing, and sequencing platforms can all influence microbial composition and detection (48), and microbiome analyses based on tumor-sequencing data face inherent difficulties in interpreting low-biomass signals (49).

## 3 Molecular mechanisms by which intratumoral microorganisms drive malignant progression of PDAC

The effects of intratumoral microorganisms on PDAC initiation and progression can be systematically considered across three core levels: inflammatory-immune signaling, metabolic-epigenetic regulation, and stromal remodeling-EMT. These levels are logically interconnected. Microbe-associated molecular patterns are first sensed by host pattern-recognition receptors, triggering inflammatory signaling cascades; inflammatory signals and microbial metabolites then converge on metabolic and epigenetic networks in tumor cells to reshape cell fate; downstream effects ultimately manifest as changes in tissue architecture and acquisition of invasive and metastatic phenotypes through stromal-cell activation and EMT. The pathogenic mechanisms and diagnostic and therapeutic value of the intratumoral microbiome are being progressively defined (50). The evidence supporting these three axes differs in source: human PDAC specimens establish microbial detection and selected clinical associations, whereas causal evidence for many inflammatory, metabolic, and stromal mechanisms still comes chiefly from animal or cell models, sometimes extrapolated from gut studies. **Figure 2** is a conceptual schematic of representative interactions rather than a complete inventory of mechanisms. **Table 2** lists the pathways discussed in this section together with the experimental system in which each was directly examined.

**Figure 2:**
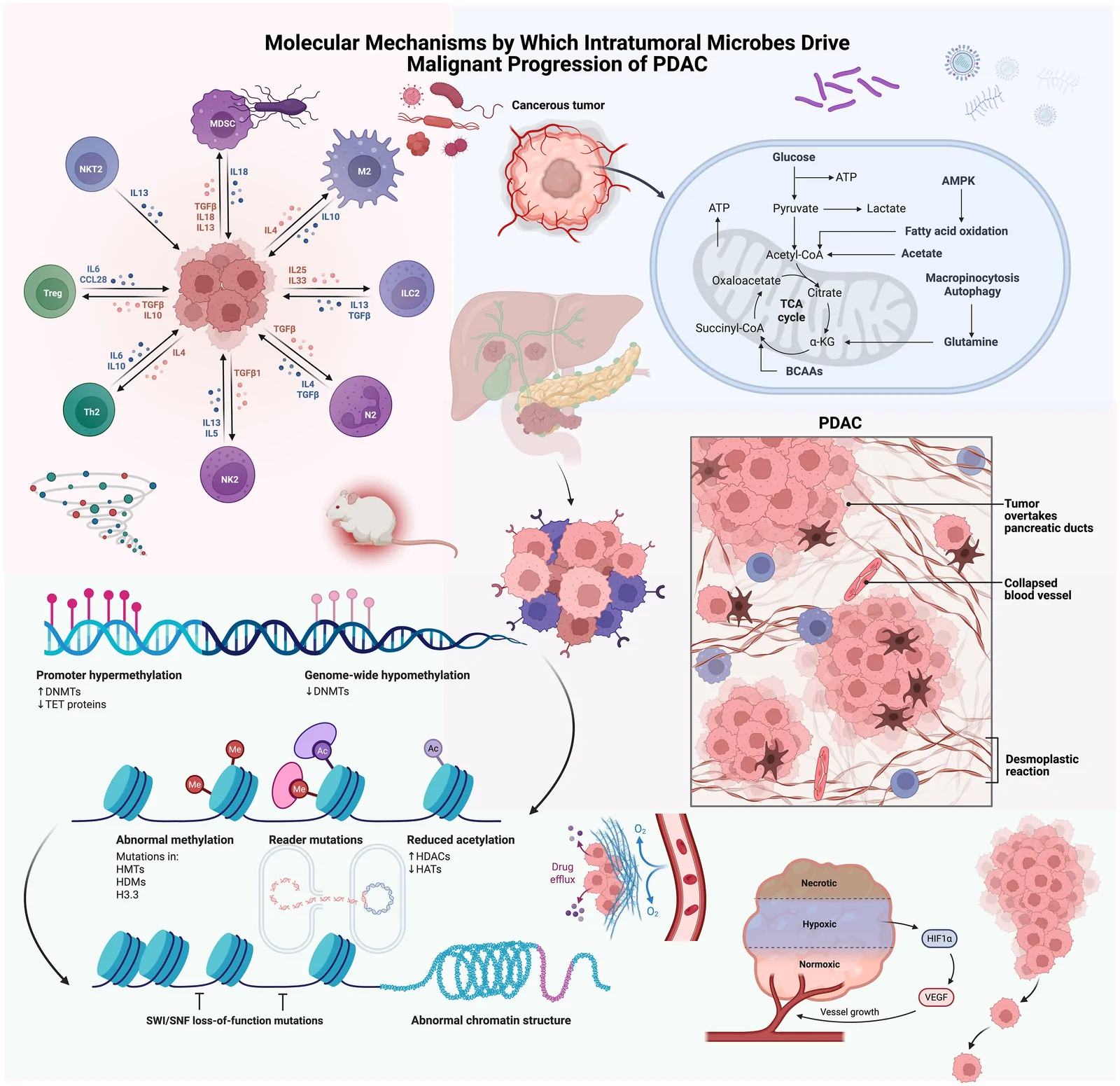
Molecular mechanisms by which intratumoral microorganisms promote the initiation and progression of PDAC. Intratumoral microorganisms contribute to the development and malignant progression of PDAC through multiple interconnected mechanisms involving immune regulation, metabolic reprogramming, epigenetic remodeling, stromal alteration, and adaptation to hypoxia. Microbial components and microbe-derived signals can reshape the tumor immune microenvironment by modulating the recruitment, polarization, and functional states of myeloid-derived suppressor cells, macrophages, regulatory T cells, T helper cells, natural killer cells, and innate lymphoid cells. These changes are accompanied by altered cytokine networks, including transforming growth factor beta, interleukin 4, interleukin 6, interleukin 10, interleukin 13, interleukin 18, interleukin 25, interleukin 33, and C-C motif chemokine ligand 28, thereby promoting immunosuppression and tumor immune escape. Intratumoral microbes may also affect central carbon metabolism and nutrient utilization by regulating glycolysis, fatty acid oxidation, glutamine metabolism, branched-chain amino acid metabolism, the tricarboxylic acid cycle, autophagy, and macropinocytosis. In parallel, microbe-associated signals can influence DNA methylation, histone modification, chromatin organization, and epigenetic regulatory enzymes, resulting in persistent transcriptional reprogramming of tumor cells. Moreover, microbial activity may enhance hypoxia-related signaling, angiogenesis, drug efflux, desmoplastic reactions, vascular compression, and pancreatic duct invasion. Collectively, these processes establish a tumor-supportive ecological niche that facilitates PDAC growth, invasion, immune evasion, metabolic plasticity, and therapeutic resistance. Human PDAC cohorts provide mainly associations; mouse models and PDAC cell lines provide experimental functional findings; and gut-derived or nonpancreatic systems provide evidence requiring pancreatic validation. The mechanisms shown differ substantially in the strength of the supporting evidence. Inflammatory and immune mechanisms, including Toll-like receptor-dependent sensing of bacterial LPS and the resulting NF-kappaB-dependent inflammatory program, are supported by experiments in mouse models and PDAC cell lines and, in human tissue, mainly by associative observations from retrospective cohorts. The metabolic, epigenetic, and stromal mechanisms are supported largely by PDAC cell lines, mouse models, or extrapolation from gut-microbiome studies, and the fungal mechanisms are supported by mouse models and PDAC cell lines, with fungal biomass in human PDAC tissue extremely low and their human relevance still debated.

**Table 2:** Molecular mechanisms by which intratumoral microorganisms drive malignant progression of PDAC.

Microorganism/metabolite	Molecular pathway	Target cell/effect	Consequence for PDAC	Evidence source
Gammaproteobacterial lipopolysaccharide (LPS)	Toll-like receptor 4 (TLR4)-nuclear factor kappa B (NF-kappaB) signaling (context-dependent adaptor effects)	Tumor and immune cells (experimental systems)	Experimental inflammation; adaptor inhibition can intensify dendritic-cell-T helper 2-driven progression	Mouse models and cell studies; human PDAC associations do not establish signaling direction
LPS/peptidoglycan	Toll-like receptor-transforming growth factor beta-Smad2/3	Pancreatic stellate cells	Pancreatic stellate cell activation and fibrotic-stroma formation	Preclinical stellate-cell/mouse studies; human pancreatic-tissue associations
Fusobacterium nucleatum Fap2	galactose-N-acetylgalactosamine binding, leading to granulocyte-macrophage colony-stimulating factor/C-X-C motif chemokine ligand 1 secretion	PDAC cells	Enhanced proliferation, migration, and invasion (independent of immune cells)	PDAC cell lines
Fusobacterium nucleatum LPS	TLR4-M2 polarization	Macrophages	Increased M2-like tumor-associated macrophages and immunosuppression	PDAC cell lines and mouse models
Anaerobic bacteria (Bacteroides/Peptoniphilus)	Independent of driver-gene mutations	cluster of differentiation 4-positive/CD8-positive T cells	Reduced T-cell infiltration and immune escape	Human PDAC cohorts (association)
Malassezia globosa beta-glucan	Dectin-1-Src/Syk-CARD9-NF-kappaB, leading to interleukin 33 secretion	T helper 2 cells/group 2 innate lymphoid cells	Type 2 immune polarization and suppression of CD8-positive T-cell function	Mouse models and PDAC cell lines; human relevance debated
Malassezia globosa mannan	mannose-binding lectin-complement lectin pathway (via associated serine proteases 1 and 2)	Tumor cells	C3a/C5a-receptor-mediated proliferation and EMT	Mouse models and PDAC cell lines; human relevance uncertain
Butyrate from butyrate-producing bacteria	histone deacetylase inhibition, leading to p21/caspase-3 activation	Colonic epithelial/colorectal-cancer cells (not PDAC)	Histone deacetylase inhibition and apoptosis in nonpancreatic cells; no proven direct effect in PDAC	Colonic/colorectal cell experiments; unvalidated in PDAC
Butyrate	Regulation of T-cell-receptor signaling	CD8-positive T cells	Enhanced response to programmed cell death protein 1 blockade in experimental models	Preclinical tumor/immune models; not demonstrated as a clinical PDAC effect
Blautia-derived 6-hydroxyhexanoic acid	Janus kinase 1-signal transducer and activator of transcription 1	Macrophages	M2-like macrophage reprogramming toward a classically activated phenotype and activation of the immune microenvironment	Human PDAC samples (association) and orthotopic mice (function)
deoxycholic acid from secondary-bile-acid-producing bacteria	epidermal growth factor receptor/mitogen-activated protein kinase/signal transducer and activator of transcription 3	PDAC cells	Promotion of proliferation	PDAC cell experiments; associative human tissue observations do not demonstrate gut-to-tumor metabolite delivery
LPS	NF-kappaB/transforming growth factor beta-Smad, leading to Snail/ZEB1 induction	PDAC cells	Induction of EMT and invasive progression	PDAC cell lines

Evidence sources distinguish human associations from functional findings in mouse, cell, or nonpancreatic models. Associations do not establish causality.

### 3.1 Microbial regulation of inflammatory and immune signaling

In experimental systems, bacterial microbe-associated molecular patterns engage distinct Toll-like receptors: LPS engages TLR4, peptidoglycan and lipoteichoic acid engage Toll-like receptor 2, and flagellin engages Toll-like receptor 5; these ligands are not all specific to Gammaproteobacteria (51, 52). In mouse and cell-based studies, microbiota-associated Toll-like receptor signaling can promote tumor growth (53) and activate the NF-kappaB transcription factor through myeloid differentiation primary response 88-dependent pathways, increasing proinflammatory mediators including interleukin 6, interleukin 8, tumor necrosis factor alpha, C-C motif chemokine ligand 2, and C-X-C motif chemokine ligand 1 and thereby establishing a persistent chronic inflammatory microenvironment (54). In oncogenic Kras-driven mouse models, Ochi et al. found that LPS-mediated TLR4 stimulation accelerated neoplastic progression, but its downstream signaling branches had opposite effects: blockade of the TIR-domain-containing adapter-inducing interferon beta-dependent pathway was protective, whereas inhibition of myeloid differentiation primary response 88 enhanced dendritic-cell-mediated T helper 2 polarization and exacerbated pancreatic carcinogenesis (55). Microbe-driven inflammatory signaling also shows molecular synergy with Kirsten rat sarcoma viral oncogene homolog (KRAS) mutation, the central oncogenic driver of PDAC. More than 90% of PDACs harbor gain-of-function KRAS mutations, most commonly G12D or G12V, which maintain RAS in a guanosine triphosphate-bound active state and constitutively activate RAF-MEK-extracellular signal-regulated kinase and phosphoinositide 3-kinase-mechanistic target of rapamycin signaling (56, 57). In preclinical PDAC systems, bacterial microbe-associated molecular patterns can activate RAS/mitogen-activated protein kinase signaling through Toll-like receptors and superimpose this signal on already elevated extracellular-signal-regulated kinase activity, further reinforcing proliferative and antiapoptotic transcriptional programs (58). In germ-free Kras/Trp53/Pdx1-Cre mice, comparative experiments support a causal contribution of microbial communities in promoting progression from pancreatic intraepithelial neoplasia to PDAC. Experimental evidence for TLR4-associated inflammation comes primarily from mouse and cell systems; the divergent effects of TIR-domain-containing adapter-inducing interferon beta and myeloid differentiation primary response 88 perturbation prohibit treating all downstream signaling as uniformly tumor promoting. The corresponding human PDAC evidence remains largely associative. In preclinical PDAC systems, Fusobacterium nucleatum-associated TLR4 stimulation can promote M2-like tumor-associated macrophage polarization; the magnitude of this effect in patients remains unestablished. In a retrospective cohort of 162 resected PDACs, Abe et al. demonstrated that intratumoral bacterial positivity was associated with denser M2-like tumor-associated macrophage infiltration and poorer overall survival and identified a significant genome-microbiome interaction between bacterial presence and KRAS mutation status (59). Based largely on experimental studies, a systematic review by D’Antonio et al. summarized proposed tumor-promoting mechanisms of F. nucleatum in PDAC, including recruitment of myeloid-derived suppressor cells through the C-X-C motif chemokine ligand 1-C-X-C motif chemokine receptor 2 axis with suppression of CD8-positive T-cell function and tumor-selective colonization through Fap2 binding to galactose-N-acetylgalactosamine residues (60, 61). In preclinical studies, intratumoral fungi have also been implicated in immunomodulatory pathways that may be partly independent of bacterial communities. In Kras/Trp53/Pdx1-Cre mice and PDAC cell lines, Alam et al. proposed a mechanism by which Malassezia globosa may promote immunosuppression: beta-glucans in the fungal cell wall are recognized by Dectin-1 receptors on PDAC cells, activating a Src/Syk/CARD9/NF-kappaB cascade that drives abundant interleukin 33 secretion (62). These mouse-model and PDAC-cell-line findings do not establish an equivalent causal role for this fungal pathway in human PDAC tissue (62). In those models, secreted interleukin 33 recruits and activates T helper 2 cluster of differentiation 4-positive T cells and group 2 innate lymphoid cells in the tumor microenvironment; these cells produce interleukin 4, interleukin 5, and interleukin 13, establishing a type 2 immune milieu that suppresses CD8-positive T-cell function. Oncogenic KRAS G12D can likewise upregulate interleukin 33 expression, and fungal signaling amplifies activity along this axis. In PDAC mouse models, amphotericin B treatment reduced tumor growth and intratumoral infiltration by T helper 2 cells and group 2 innate lymphoid cells. In parallel with the Dectin-1-interleukin 33 pathway, M. globosa can also exert tumor-promoting effects through the lectin pathway of complement activation. In mouse-model experiments, Aykut et al. (2019) reported that mannan ligands on the surface of M. globosa are recognized by mannose-binding lectin, activating the complement lectin pathway through mannose-binding lectin-associated serine proteases 1 and 2, producing C3b deposition on tumor cells and triggering C3a/C5a-receptor-mediated signals that promote proliferation and EMT (17). In mice, genetic deficiency of mannose-binding lectin or complement C3 slowed tumor progression despite M. globosa exposure; in human transcriptomic data, higher MBL2 expression was associated with shorter PDAC survival, a correlation that does not establish fungal causality (63). Fungal effects in PDAC are strongly species specific: in recolonization experiments, only Malassezia accelerated tumor growth in mice, whereas Candida, Saccharomyces, and Aspergillus did not produce similar effects (17), indicating that fungi as a domain cannot be equated collectively with tumor-promoting activity. Candida, a common fungus of the intestinal and biliary systems, is significantly enriched in patients with chronic pancreatitis (27); its intratumoral colonization level and functional role in PDAC remain concrete questions for future investigation. Tumor-associated fungi may also indirectly influence tumor and immune cells through nutrient competition and metabolite secretion, constituting a tripartite nutrient-fungus-host interaction (64). In summary, animal and cell-based experiments support links between bacterial pattern recognition, KRAS-associated signaling, macrophage polarization, and tumor progression, while retrospective human PDAC cohorts support selected microbial-immune-prognosis associations. In mouse models and PDAC cell lines, M. globosa has been linked to Dectin-1-interleukin 33 and mannose-binding lectin-complement signaling; the relevance of these proposed fungal immune-evasion pathways to humans remains debated. The applicability of fungal mechanisms to human PDAC depends on reliable estimation of intratumoral fungal abundance and clarification of causal status. Aykut et al. proposed substantial fungal enrichment in PDAC on the basis of sequencing data from human pancreatic tissue, whereas an independent reanalysis using a different bioinformatic workflow failed to reproduce differences in fungal communities between normal pancreas and PDAC and highlighted limitations in the reliability of fungal signals in low-biomass samples (28). The original investigators responded from the perspectives of methodological design and functional validation (29). This methodological disagreement directly affects how fungal involvement should be classified. Recent reviews suggest that tumor-associated fungi may more often act as opportunistic bystanders within the microenvironment rather than as universally oncogenic microorganisms, and extrapolation of tumor-promoting effects to human PDAC therefore requires caution (65, 66). For M. globosa, the key functional evidence supporting a tumor-promoting role-including fungal ablation, single-species recolonization, and delayed tumor growth after antifungal treatment-was generated in mouse models, with related cellular experiments limited to PDAC cell lines (66, 67). Fungal biomass in human PDAC tissue is extremely low, and establishing a causal role requires direct validation in human specimens; this is a priority before fungal mechanisms can be considered clinically. The roles of other fungi such as Candida and Aspergillus in PDAC, as well as the net effect of fungal-bacterial interactions in the tumor microenvironment, remain to be resolved despite evidence for their presence.

### 3.2 Microbial regulation of metabolic and epigenetic pathways

Short-chain fatty acids are candidate mediators of microbiome-associated effects on PDAC, but direct intratumoral exposure and causal effects in patients remain uncertain. In normal colonic epithelial cells, butyrate serves as a major energy source and is consumed through beta-oxidation. In colonic and colorectal cancer cell models displaying the Warburg effect, however, restricted beta-oxidation causes intracellular accumulation of butyrate, which acts as a histone-deacetylase inhibitor, induces histone hyperacetylation, activates p21/WAF1 expression and caspase-3-dependent apoptosis, and suppresses NF-kappaB activation through G protein-coupled receptor 109A signaling (68–70). These pathways have been defined mainly in colonic epithelial and colorectal-cancer cells; direct effects in human PDAC tissue still require validation (71). In experimental immune-cell systems, butyrate and propionate can regulate immune-cell function through G protein-coupled receptor 41 and G protein-coupled receptor 43 (72–74). Mouse experiments also suggest antitumor activity for butyrate: in PDAC mouse models, butyrate supplementation not only inhibits tumor proliferation and enhances responsiveness to gemcitabine but also preserves intestinal barrier integrity by alleviating chemotherapy-induced mucositis and reducing intestinal permeability, thereby decreasing endotoxin translocation to the pancreas and indirectly suppressing TLR4-mediated tumor-promoting inflammation (45, 46). Short-chain fatty acids also regulate antitumor immunity in gastrointestinal malignancies. The short-chain fatty acid receptors G protein-coupled receptor 41 and G protein-coupled receptor 43 are regarded as potential therapeutic targets in several diseases and cancers; their expression patterns and signaling functions in PDAC are key parameters for determining the applicability of this strategy (75). In human PDAC samples and complementary mouse experiments, Zhong et al. investigated an intratumoral bacterial metabolite: enrichment of Blautia in PDAC tissue was associated with better prognosis, and its metabolite 6-hydroxyhexanoic acid, a medium-chain fatty acid, activated Janus kinase 1-signal transducer and activator of transcription 1 signaling to reprogram M2-like tumor-associated macrophages toward a classically activated phenotype, shifting the immune microenvironment from suppressive to activated (76). This finding was based on 16S ribosomal ribonucleic acid sequencing and metabolomic analysis of samples from patients with PDAC and was functionally validated in an orthotopic PDAC mouse model, providing the first evidence for a role of a medium-chain fatty acid in PDAC immune regulation. Systematic determination of short-chain fatty acid receptor expression and effective intratumoral metabolite concentrations in PDAC is essential for establishing whether these metabolites can act directly within the tumor microenvironment.

In gut-derived metabolic pathways, secondary bile acids provide another potential connection with PDAC. Intestinal bacteria carrying 7alpha-dehydroxylase genes can convert primary bile acids into secondary bile acids, among which deoxycholic acid can promote PDAC-cell proliferation in cell-based experiments by activating epidermal growth factor receptor, mitogen-activated protein kinase, and signal transducer and activator of transcription 3 pathways (47). In contrast, acetate derived from cancer-associated fibroblasts drives metabolic adaptation in PDAC through acetyl-coenzyme A synthetase 2-dependent histone acetylation. In primary PDAC stromal-cell experiments and complementary mouse studies, Murthy et al. showed that cancer-associated fibroblasts metabolize citrate to acetate through ATP citrate lyase and secrete acetate extracellularly. After uptake by PDAC cells, acetate is converted to acetyl-coenzyme A by acetyl-coenzyme A synthetase 2; acetyl-coenzyme A synthetase 2-dependent H3K27 acetylation promotes acetylation of the specificity protein 1 transcription factor at lysine 19, enhancing specificity protein 1 stability and transcriptional activity and upregulating spermidine/spermine N1-acetyltransferase 1, thereby promoting PDAC-cell survival in an acidic microenvironment through polyamine-homeostasis reprogramming (77). These two metabolic pathways are supported by different types of evidence. The cancer-associated fibroblast-acetate axis has been functionally validated in primary PDAC stromal cells and gene-knockout mice, whereas the proliferative effect of deoxycholic acid is currently supported mainly by cell-based experiments and associative evidence. Deoxycholic acid is generated predominantly by gut bacteria in the colon; its transport efficiency to pancreatic tissue and the intratumoral microenvironment, together with the local effective concentration achieved, are necessary parameters for assessing the actual weight of this pathway in PDAC. The host-stromal metabolic pathway represented by the cancer-associated fibroblast-acetate axis and the butyrate effects of butyrate-producing bacteria act in opposing directions. Their coexistence indicates bidirectional metabolic regulation within the tumor microenvironment and underscores the need to distinguish microbial from host sources when interpreting metabolite effects (77).

### 3.3 Microbial regulation of stromal remodeling and EMT

Beyond immune-cell effects, experimental studies suggest that bacterial signals can influence PDAC stromal remodeling, but evidence for direct microbial induction of fibrosis in human tumors remains limited. In experimental stromal-cell and mouse-based systems, LPS and peptidoglycan have been implicated in activation of pancreatic stellate cells through transforming growth factor beta-Smad2/3 signaling, and activated pancreatic stellate cells upregulate connective tissue growth factor and collagen genes (COL1A1 and COL3A1), accelerating extracellular-matrix deposition (78). In the proposed preclinical feedback model, activated pancreatic stellate cells may further amplify inflammatory signaling through paracrine interleukin 6 and C-X-C motif chemokine ligand 12, establishing a positive-feedback loop of microbial stimulation, pancreatic stellate cell activation, fibrosis, inflammation, and further pancreatic stellate cell activation (79, 80). In established PDAC stromal biology, desmoplasia impedes drug penetration and can exclude effector T cells from tumors through the C-X-C motif chemokine ligand 12-C-X-C motif chemokine receptor 4 axis, thereby providing a stromal basis for immune escape and chemoresistance. In cell-based experiments, microbial stimulation can also enhance PDAC-cell migration and invasion through EMT. PDAC cell lines exposed to LPS display characteristic EMT changes, including downregulation of E-cadherin and upregulation of vimentin, Snail, and Twist, accompanied by increased secretion of matrix metalloproteinase-2 and matrix metalloproteinase-9 (21). In those LPS-treated PDAC cell lines, the proposed mechanism involves cooperation between NF-kappaB and transforming growth factor beta signaling: NF-kappaB directly induces transcription of Snail and ZEB1, whereas transforming growth factor beta promotes formation of a transcriptional cooperation complex between Smad proteins and Snail that jointly suppresses the E-cadherin promoter. Preclinical oncogenic KRAS/tumor protein p53 systems suggest enhanced susceptibility to microbe-associated EMT cues, forming an amplification cascade from genetic susceptibility to microbial triggering and invasive progression (81). This EMT phenotype has so far been demonstrated in PDAC cell lines after exogenous LPS stimulation; defining the spatial relationship between microbial exposure and EMT markers in human PDAC tissue is a key step toward establishing its relative importance *in vivo*. In addition to LPS-stimulation experiments, cell-line data implicate Fusobacterium nucleatum in increased PDAC-cell migration and invasion through its adhesin system. In multiple PDAC cell lines, Udayasuryan et al. demonstrated that F. nucleatum can enter host cells through binding of the Fap2 surface adhesin to galactose-N-acetylgalactosamine residues. Infected PDAC cells secrete granulocyte-macrophage colony-stimulating factor, C-X-C motif chemokine ligand 1, interleukin 8, and macrophage inflammatory protein 3 alpha, which enhance proliferation and invasive motility of neighboring uninfected PDAC cells through autocrine and paracrine signaling (82). This effect can occur independently even in the absence of immune and stromal cells. In the tested cell-based system, multiple F. nucleatum subspecies, including nucleatum, animalis, vincentii, and polymorphum, exhibited this function. This work establishes a direct model independent of immune and stromal cells; bacterial inoculum, exposure duration, and *in vivo* accessibility form the quantitative basis for translating this model to the intratumoral setting. In summary, experimental pancreatic stellate cell and mouse models suggest bacteria-linked fibrosis, whereas LPS-stimulated and F. nucleatum-exposed PDAC cell lines support EMT or invasive phenotypes. Whether these pathways operate at clinically meaningful magnitudes in human tumors requires spatial colocalization, quantitative exposure measurements, and human-derived functional validation.

## 4 Intratumoral microorganisms and therapeutic response in PDAC

PDAC is a malignancy with poor prognosis. Surgical resection remains the principal treatment for resectable PDAC, but recurrence is common even after radical resection (83). Conventional multimodal treatment includes chemotherapy, radiotherapy, immunotherapy, and targeted therapy, yet efficacy remains limited. The microbiome exerts multilayered regulatory effects on PDAC treatment (84), and organoid models can be used to validate microbe-drug interactions (85). In recent years, strategies based on intestinal-microecology theory have provided new ideas for PDAC treatment. Intratumoral microorganisms can not only directly alter chemotherapy efficacy through enzymatic drug inactivation but also influence immune-checkpoint-inhibitor responses by remodeling the tumor immune microenvironment and show potential value as biomarkers for prognostic stratification. These three roles are linked along the translational pathway: the clinical value of microbiome information first depends on identifying patients carrying specific microbe-driven mechanisms, whereas chemoresistance and immunotherapy failure arise, respectively, from direct microbial drug inactivation and indirect microbial remodeling of the immune microenvironment. This mechanistic distinction determines the appropriate patient populations and optimal sequencing for corresponding interventions. This section therefore examines the relationship between intratumoral microorganisms and PDAC treatment response, together with its translational prospects, across five dimensions: biomarkers, chemoresistance, immune response, enhancement of efficacy while limiting toxicity, and microbiome-targeted therapy. **Figure 3** illustrates the microbiome-dependent modulation of therapeutic response and the candidate microbiome-directed interventions, and **Table 3** summarizes the corresponding therapeutic strategies.

**Figure 3:**
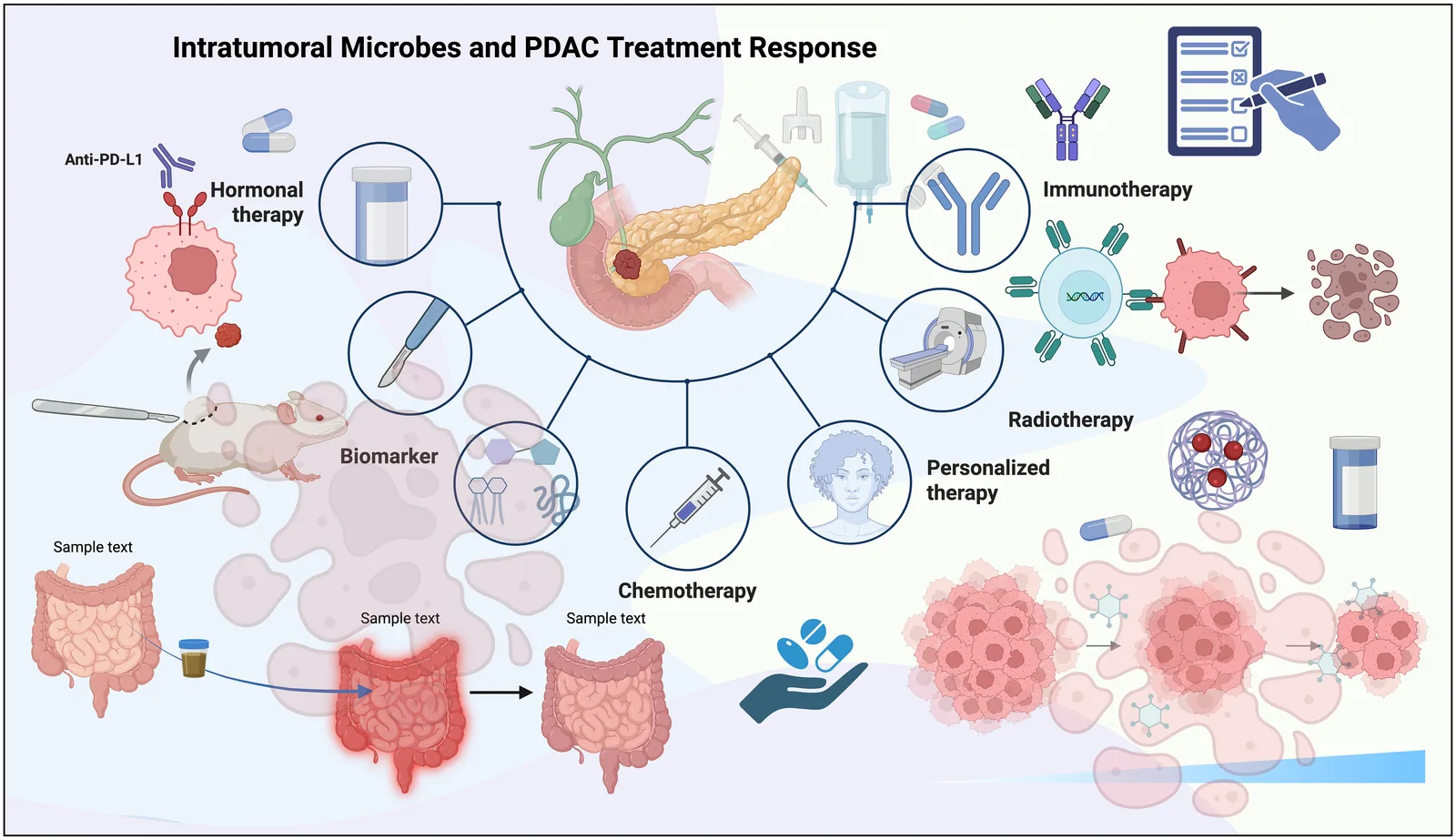
Therapeutic strategies targeting intratumoral microorganisms in PDAC. Intratumoral microorganisms are under investigation as possible therapeutic targets and candidate biomarkers for improving the management of PDAC. Profiling microbial composition, abundance, spatial distribution, and functional activity in tumor tissues, body fluids, or intestinal samples may provide clinically relevant information for patient stratification, prognosis assessment, treatment selection, and prediction of therapeutic response. Microbiota-directed interventions may include selective depletion of tumor-associated microorganisms, suppression of microbial metabolic activity, modulation of microbial communities, or restoration of a more favorable host-microbe ecosystem. Potential approaches include antimicrobial agents, bacteriophage-based strategies, microbiome-modulating interventions, and targeted manipulation of specific microbial products or signaling pathways. These approaches may be integrated with conventional chemotherapy, radiotherapy, immunotherapy, and other systemic treatments to reduce microorganism-mediated drug inactivation, alleviate immunosuppressive signaling, and enhance antitumor immune responses. Intratumoral microbial signatures may also serve as biomarkers for identifying patients who are more likely to benefit from particular treatment modalities. In addition, combined analysis of microbial features, tumor molecular characteristics, host immune status, and treatment response may support the development of personalized therapeutic strategies. Such microbiome-informed approaches provide a conceptual framework for converting intratumoral microbial heterogeneity from a contributor to therapeutic resistance into a clinically exploitable determinant of precision treatment in PDAC. The strategies shown differ substantially in the strength of the supporting evidence and in their stage of development. Antimicrobial depletion of drug-resistance-associated bacteria is supported by human intratumoral isolates, retrospective clinical associations, and mouse experiments, whereas engineered probiotics, postbiotics, phage-based approaches, and dietary or metabolite-directed interventions are supported mainly by animal and cell-based studies and remain at the preclinical proof-of-concept stage. The image is a conceptual overview of reported strategies and is not intended to indicate that any of them has established clinical efficacy in patients with PDAC.

**Table 3:** Therapeutic intervention strategies targeting the intratumoral microbiome in PDAC.

Strategy category	Specific approach	Target	Research stage	Major limitation
Antibiotics	Broad-spectrum antibiotics (ciprofloxacin/metronidazole)	Eliminate CDD_L-producing Gammaproteobacteria and restore gemcitabine activity	Retrospective human associations and mouse interventions	Disruption of intestinal commensal communities; increased risk of resistance
Narrow-spectrum antimicrobials and bacteriophages	Targeted bacteriophages; nuclease-programmed phages	Selectively eliminate resistant bacteria or edit the CDD_L gene	Preclinical proof of concept	Limited delivery efficiency; risk of target-bacterium resistance evolution; unclear regulatory pathway
Engineered probiotics	Gallium-polyphenol-functionalized Lactobacillus rhamnosus GG	Eliminate intratumoral Proteobacteria, reduce TLR4 activation, and enhance anti-programmed death-ligand 1 efficacy	Orthotopic PDAC mouse models	Insufficient data on human safety and colonization stability
Postbiotics	Butyrate/valerate supplementation; Lactobacillus-derived ferrichrome	histone deacetylase inhibition and immune regulation; TLR4-dependent reprogramming of tumor-associated macrophages from M2-like toward classically activated states	Animal and *in vitro* experiments	Dose, route of administration, and intratumoral exposure have not been optimized; clinical translation pathway remains unclear
Dietary intervention	Resistant starch/dietary fiber	Enrich butyrate-producing bacteria and increase local short-chain fatty acid levels	Animal experiments	Insufficient PDAC-specific clinical evidence
Microbiota transplantation	Fecal microbiota transplantation from long-term survivors	Reconstruct a protective intratumoral microbiome and increase CD8-positive T-cell infiltration	Mouse fecal microbiota transplantation experiments	Infection risk; fatal bacteremia has been reported

The six strategies shown are at different stages of development. Retrospective associations are not evidence of clinical efficacy, and most microbiome-directed interventions remain preclinical. Broad-spectrum antibiotics and fecal microbiota transplantation require particular attention to microbiome disruption and infectious risks.

### 4.1 Biomarkers

Riquelme et al. identified Saccharopolyspora, Pseudoxanthomonas, Streptomyces, and Bacillus clausii as taxa associated with favorable prognosis in PDAC (22). Microbiome composition is also significantly associated with prognosis in Chinese PDAC cohorts (86, 87), and Mendelian-randomization analyses support a causal effect of the gut microbiota on PDAC (15). Fecal microbiota transplantation experiments provide additional directional causal evidence: tumor-bearing mice receiving fecal microbiota from long-term-surviving patients developed significantly smaller tumors than mice receiving fecal microbiota from short-term survivors, and the former group showed greater CD8-positive T-cell infiltration in the tumor microenvironment (22). In an independent Japanese cohort, Abe et al. (2024) confirmed the prognostic value of anaerobic bacterial communities as an independent factor, supporting the cross-population relevance of this biomarker concept (59). In an independent validation cohort of Chinese patients with PDAC, the long-term-survivor-associated taxa differed substantially from those identified in the United States cohort, suggesting that host genetic background and dietary habits are important determinants of the PDAC intratumoral microbial niche and that the transferability of prognostic models across populations requires systematic validation in multicenter international cohorts. Sources of cross-cohort variation span host genetics, dietary structure, and analytical procedures. Polymorphisms in immune-related genes may alter selective pressures governing microbe-host interactions; differences in dietary fiber and polyphenol intake can change the composition of fermentable substrates in the gut and thereby alter the taxa that become enriched; collection and storage conditions, DNA-extraction methods, and sequencing platforms can change estimates of community composition and diversity; and whether tumor purity, sequencing depth, and batch effects are adjusted can alter the magnitude and even direction of prognostic associations (48). Distinguishing these technical variables from biological differences requires standardized sample processing and decontamination, prespecified analytical plans and external validation in multicenter cohorts, and stratified analyses incorporating host genetic background and dietary factors. Plasma microbial cell-free DNA has shown preliminary potential as a noninvasive liquid-biopsy signal for tumor detection and classification; its sensitivity and diagnostic thresholds for early PDAC screening require further definition in prospective human cohorts. Molecular classification of PDAC based on microbial-community composition-for example, immunosuppressive subtypes enriched in Bacteroides/Lactobacillus and poor-prognosis subtypes dominated by Pseudomonadaceae-provides an additional dimension for patient stratification (88). Joint analysis of fecal and intratumoral microbiota may provide more accurate diagnostic information (89). Measurement of circulating short-chain fatty-acid levels may also serve as a noninvasive functional biomarker of response to probiotic interventions (90).

### 4.2 Chemotherapy resistance

A direct route by which microbes alter treatment is enzymatic drug inactivation: CDD_L expressed by Gammaproteobacteria represents the best-supported functional mechanism of microbiome-mediated chemoresistance identified to date. Using bacterial enzyme assays, genetic experiments, and tumor models, Geller et al. demonstrated that CDD_L expressed by Gammaproteobacteria, including Klebsiella, Proteus, and other taxa enriched in PDAC, can hydrolyze gemcitabine (2′,2′-difluorodeoxycytidine) *in situ* to inactive 2′,2′-difluorodeoxyuridine (9). CDD_L differs in substrate specificity from the bacterial short isoform of cytidine deaminase: gemcitabine is efficiently inactivated by CDD_L, whereas bacteria containing the short isoform generally did not confer resistance, although exceptions have been reported. This pattern supports the importance of bacterial enzyme isoform for drug resistance. In tumor-bearing mouse models, antibiotic treatment restored gemcitabine activity; corresponding clinical efficacy remains unproven. In a retrospective human cohort, Fulop et al. observed an association with improved overall survival among patients with metastatic PDAC who received antibiotics while being treated with gemcitabine-based regimens, whereas no such benefit was observed with 5-fluorouracil-based regimens, further supporting the existence of bacteria-specific gemcitabine resistance (91). Recent work has resolved this mechanism to a key intratumoral species: Peng et al. isolated Enterobacter hormaechei from PDAC tissue and demonstrated that it drives gemcitabine resistance through drug inactivation mediated by CDD_L. This finding advances bacterial gemcitabine resistance from a phylum-level concept involving Gammaproteobacteria to a specific species and intratumoral-colonization level, suggesting that clinical resistance phenotypes may be directly related to intratumoral enrichment of particular strains. Zhao et al. further identified a clinically relevant route for dissemination of this CDD_L-associated resistance mechanism: multidrug-resistant Klebsiella pneumoniae can translocate directly from the intestine to PDAC tissue, carry antibiotic-resistance genes including acrB, mdtC, and cpxA, inactivate gemcitabine through cytidine deaminase, and increase the risk of postoperative infection (34). Precision strategies targeting CDD_L-producing bacteria, such as Gammaproteobacteria-specific bacteriophages or inhibitors of CDD_L enzymatic activity, are promising but remain at the proof-of-concept stage (92). The value of the microbiome as a biomarker has been widely recognized (93). This mechanism currently represents the most complete evidence chain for a microbe-drug interaction in the field, combining enzymatic and gene-knockout mechanistic evidence, interventional validation in animal models, and indirect support from human cohorts; it therefore provides a benchmark against which other candidate mechanisms can be evaluated.

### 4.3 Immune response

Beyond drug inactivation, immune modulation constitutes a second therapeutic consideration. Current immune-checkpoint inhibitors have very limited activity in PDAC, with objective response rates below 5%; only the microsatellite-instability-high/mismatch-repair-deficient subtype, which accounts for approximately 1%-3% of PDAC, shows relatively high response rates to programmed cell death protein 1 blockade (94). Experimental data suggest several ways in which microorganisms might modify responses to immune-checkpoint inhibitors. In experimental tumor and immune systems, microbial butyrate can enhance the antitumor efficacy of anti-programmed cell death protein 1 therapy by regulating T-cell-receptor signaling (95). Mechanisms through which the gut microbiome influences immune-checkpoint blockade have been systematically summarized and include microbial antigen mimicry, regulation of T-cell differentiation and function by microbial metabolites, and activation of dendritic cells and interferon pathways (96). These mechanisms were established largely in studies of the gut microbiome and extraintestinal tumor models; their effectiveness within the PDAC intratumoral microenvironment is modified by the characteristically low immune infiltration and dense stroma of PDAC and therefore requires dedicated evaluation. Recent advances in immune-checkpoint inhibitor therapy for PDAC underscore the importance of combination treatment (97). In ex vivo immune-cell experiments, short-chain fatty acids can also improve antitumor immune responses by enhancing *in vitro* expansion and adaptation of T cells and chimeric antigen receptor T cells (98). In human PDAC cohorts, intratumoral taxa enriched among long-term survivors, including Saccharopolyspora, Pseudoxanthomonas, Streptomyces, and Bacillus clausii, were associated with immune-cell infiltration and survival. Experiments transferring survivor-associated microbiota into mice provide functional support for enhanced tumor T-cell recruitment, but the specific role of these taxa in human tumor-antigen presentation remains unproven (22). In preclinical model systems, cyclic dinucleotides synthesized by Gram-positive bacteria, including cyclic diadenosine monophosphate and cyclic diguanylate monophosphate, can activate stimulator of interferon genes signaling and drive type I interferon secretion, promoting recruitment of natural killer cells and CD8-positive T cells into the tumor microenvironment (99). The cyclic GMP-AMP synthase-stimulator of interferon genes pathway exerts bidirectional regulatory effects during cancer progression (100). In PDAC, stimulator of interferon genes signaling is further constrained by DNA methylation-mediated silencing of the stimulator of interferon genes promoter, involving DNA methyltransferase 1 (101), an epigenetic state that defines the therapeutic space for bacteria-derived stimulator of interferon genes agonists in PDAC. Inhibitors of cluster of differentiation 73 and agonists of cluster of differentiation 40 are being studied for PDAC combination therapy (102, 103); these studies do not independently validate microbiome-directed immunotherapy.

### 4.4 Enhancing efficacy while limiting toxicity

Because immune activation and infection risk may change together, adjunctive microbiome-directed interventions should prioritize strategies that can be incorporated into existing treatment frameworks without disrupting protective intestinal commensal communities. Dietary modification and prebiotic supplementation, including functional components such as resistant starch, can selectively enrich butyrate-producing bacteria such as Faecalibacterium prausnitzii and Roseburia spp. and increase intestinal butyrate availability; equivalent intratumoral exposure has not been established (104, 105). In experimental studies, butyrate supplementation can alleviate chemotherapy-induced intestinal mucositis and maintain intestinal-barrier integrity, thereby offering the potential to enhance efficacy while reducing toxicity (106). Safety remains critical because immunosuppressive chemotherapy and broad-spectrum antibiotics increase infection risk in patients with PDAC, and the United States Food and Drug Administration has reported fatal bacteremia caused by extended-spectrum beta-lactamase-producing Escherichia coli transmitted through fecal microbiota transplantation; rigorous clinical trials are therefore required. More mechanistically precise interventions do not rely on global manipulation of the microbial community (107). Precision approaches, including Gammaproteobacteria-specific bacteriophage therapy, bacteriophages programmable using clustered regularly interspaced short palindromic repeats-associated nucleases targeting the CDD_L gene, and engineered probiotic systems such as gallium-polyphenol-functionalized Lactobacillus rhamnosus GG, have shown promise (108). In an orthotopic PDAC mouse model, Han et al. showed that Lactobacillus rhamnosus GG functionalized with a gallium-polyphenol network selectively eliminated intratumoral Proteobacteria by disrupting bacterial iron respiration, reduced tumoral Toll-like receptor activation and immunosuppressive myeloid populations, and enhanced the efficacy of programmed death-ligand 1 blockade (108). These precision approaches have so far been validated in PDAC mouse models; delivery efficiency and safety in patients with human PDAC require dedicated clinical investigation.

### 4.5 Microbiome-targeted therapy

On the basis of the mechanisms above, interventions targeting the PDAC intratumoral microbiome can be systematically classified into four levels: antibiotics, probiotics, postbiotics, and precision editing. Antibiotic intervention is the most direct approach. A retrospective study by Fulop et al. suggested that antibiotic use was associated with improved survival in patients with PDAC treated with gemcitabine (91). However, broad-spectrum antibiotics may disrupt the protective immunoregulatory functions of intestinal commensal communities, and their net benefit therefore requires careful evaluation. Probiotic and postbiotic interventions may represent safer alternatives. Lactobacillus-derived ferrichrome can reprogram tumor-associated macrophages from an M2-like to a classically activated phenotype through TLR4 signaling and promote CD8-positive T-cell infiltration (109). Chaib et al. demonstrated this effect in PDAC mouse models independently of live bacteria, suggesting a potential safety advantage of postbiotics in immunocompromised patients (109). Precision editing represents a future direction. Clustered regularly interspaced short palindromic repeats-associated nuclease systems can be programmed to specifically edit the CDD_L gene, eliminating bacterial gemcitabine resistance without killing commensal bacteria (93). Kabwe et al. systematically summarized the potential application of bacteriophage therapy in PDAC and noted that Gammaproteobacteria-specific phages could selectively eliminate drug-resistant bacteria without disrupting intestinal commensal communities (92). These precision strategies remain at the preclinical proof-of-concept stage, and their *in vivo* safety, evolution of bacterial resistance, and regulatory pathways require systematic evaluation.

At present, only antimicrobial strategies targeting CDD_L have relatively clear mechanistic and animal-experimental support; most other strategies remain at the proof-of-concept or early preclinical stage and share limitations including insufficient strain specificity, unknown long-term safety, limited delivery efficiency, and unclear regulatory pathways. Current microbiome interventions should therefore be understood as candidate adjuncts to standard therapy rather than stand-alone anticancer treatments. The therapeutic logic proceeds from direct enzymatic inactivation of chemotherapy to immune-microenvironment remodeling and, ultimately, mechanism-guided combination treatment. CDD_L-directed elimination directly blocks bacterial enzymatic inactivation of a drug, and the appropriate population can in principle be defined by intratumoral enrichment of CDD_L-producing strains. In contrast, microbiome remodeling and integration strategies act indirectly through the immune microenvironment, and their efficacy depends on whether a tumor retains an immune foundation that can be reactivated. Accordingly, rational combination strategies should first identify the relevant microbe-drug or microbe-immune interaction and then select a matched intervention and treatment sequence. This framework may also help explain inconsistent results across studies of antibiotics or probiotics: without mechanism-based enrollment criteria, a potentially responsive subgroup can be diluted by patients for whom the intervention is not applicable. Establishing practical stratification criteria and converting microbiome information into an input for treatment decisions are therefore central to clinical translation in this field.

## 5 Conclusions and future perspectives

Intratumoral microorganisms are increasingly associated with PDAC initiation, progression, and treatment resistance, although the causal strength of individual mechanisms varies. At the inflammatory-immune level, mouse and cell studies implicate Gammaproteobacterial LPS-TLR4-associated inflammation in KRAS-driven progression, with context-dependent downstream adaptor signaling. Experimental Fusobacterium nucleatum exposure has been linked to TLR4-associated M2-like polarization. Human cohort studies associate selected anaerobic communities with reduced T-cell infiltration independently of driver-gene mutations without establishing causation. In mouse and cell models, Malassezia globosa has been linked to proposed immune-evasion pathways involving Dectin-1-interleukin 33 signaling with T helper 2 and group 2 innate lymphoid cell responses and the mannose-binding lectin-complement pathway. At the metabolic-epigenetic level, in colonic and colorectal cell models, butyrate can inhibit histone deacetylases and induce apoptosis, although a corresponding direct mechanism in human PDAC has not been established. Human PDAC observations and complementary mouse experiments link Blautia-derived 6-hydroxyhexanoic acid to macrophage reprogramming from an alternatively activated phenotype toward a classically activated phenotype through Janus kinase 1-signal transducer and activator of transcription 1 signaling. Experimental studies suggest that deoxycholic acid from secondary-bile-acid-producing bacteria can promote proliferation through epidermal growth factor receptor/mitogen-activated protein kinase/signal transducer and activator of transcription 3 signaling. Experimental work suggests that cancer-associated fibroblast-derived acetate can promote PDAC-cell survival through the acetyl-coenzyme A synthetase 2-specificity protein 1-spermidine/spermine N1-acetyltransferase 1 axis independently of microbial metabolism. At the level of stromal remodeling and EMT, experimental Gammaproteobacterial signals may promote fibrotic-stroma formation through the pancreatic stellate cell-transforming growth factor beta axis and induce EMT through LPS-NF-kappaB/transforming growth factor beta signaling, whereas PDAC cell-line experiments implicate F. nucleatum in tumor-cell invasion through the Fap2-linked granulocyte-macrophage colony-stimulating factor and C-X-C motif chemokine ligand 1 signaling. In therapeutic regulation, experimentally demonstrated *in situ* enzymatic inactivation of gemcitabine by Gammaproteobacterial CDD_L is currently the best-supported microbiome-mediated mechanism of chemoresistance, while long-term-survivor-associated microbial signatures remain prognostic associations, and engineered probiotic systems are preclinical candidates for immunotherapy sensitization.

These mechanisms are not supported by equivalent levels of evidence. For bacterial gemcitabine inactivation, human intratumoral isolates and clinical associations complement enzyme assays and mouse interventions. Human PDAC cohorts also support associations between microbial features, immune infiltration, and survival, but do not establish the causal magnitude of inflammatory signaling in patients. By contrast, functional evidence for proposed fungal signaling pathways derives mainly from mouse models and PDAC cell lines; the independent reanalysis failed to reproduce a consistent human-tissue fungal signal, and human causal relevance remains debated; fungal biomass in human PDAC tissue is extremely low, and the functional importance of fungi in humans is a priority question for future research. A central issue in current PDAC intratumoral-microbiome research is reproducibility across studies, because resolution of this issue directly determines the reliability of conclusions in the field. Total bacterial biomass in PDAC tissue is extremely low and may be of the same order of magnitude as background contamination from reagents and consumables, making standardized quality-control procedures essential for data reliability. Most existing human cohort studies are cross-sectional or retrospective and therefore provide associative rather than causal evidence. Causal validation in animal models, including germ-free colonization and fecal microbiota transplantation experiments, provides directional evidence, but differences between mouse and human pancreatic microbial communities limit interspecies extrapolation. Differences in long-term-survivor-associated taxa between United States and Chinese cohorts indicate that host genetic background and dietary habits materially influence the PDAC microbial niche. Evidence regarding fungi is dominated by mouse models and cell lines: the key functional experiments supporting a tumor-promoting role of Malassezia were performed mainly in these systems, whereas fungal biomass in human PDAC tissue is extremely low and results are inconsistent across studies. Independent reanalysis of original sequencing data failed to reproduce intratumoral fungal-community differences, indicating that interpretation of fungal signals is highly dependent on sample processing and bioinformatic workflows. Systematic progress along these lines may transform the intratumoral microbiome from a bystander in disease-mechanism studies into a driver of precision diagnosis and treatment in PDAC.

Future research directions. Technical priorities include reproducible identification of microbes in low-biomass pancreatic tissue through standardized sampling, negative controls, contamination management, and independent quantification; species- and strain-level functional testing in human-derived organoid, immune-cell, and stromal systems, including independent confirmation of the disputed fungal findings; and spatial and single-cell approaches that distinguish organisms located within tumors from gut-derived signals and that map the local host response. Clinical priorities concern multicenter prospective validation of microbial and treatment-response biomarkers across patient populations, and carefully designed interventional studies to determine whether microbiome-directed approaches improve outcomes when combined with established PDAC therapies. Such studies should assess treatment benefit alongside microbiome disruption, infection risk, and reproducibility. Progress on these priorities may clarify which observations can support clinical decisions, without assuming that every experimental mechanism translates directly to patients.

## References

[1] Torre LA Siegel RL Ward EM Jemal A . Global cancer incidence and mortality rates and trends--an update. Cancer Epidemiol Biomarkers Prev. (2016) 25:16–27. doi: 10.1158/1055-9965.EPI-15-0578 26667886

[2] Khalaf N El-Serag HB Abrams HR Thrift AP . Burden of pancreatic cancer: From epidemiology to practice. Clin Gastroenterol Hepatol. (2021) 19:876–84. doi: 10.1016/j.cgh.2020.02.054 32147593 PMC8559554

[3] Ho WJ Jaffee EM Zheng L . The tumour microenvironment in pancreatic cancer - clinical challenges and opportunities. Nat Rev Clin Oncol. (2020) 17:527–40. doi: 10.1038/s41571-020-0363-5 32398706 PMC7442729

[4] Bear AS Vonderheide RH O’Hara MH . Challenges and opportunities for pancreatic cancer immunotherapy. Cancer Cell. (2020) 38:788–802. doi: 10.1016/j.ccell.2020.08.004 32946773 PMC7738380

[5] Li Y Xiang S Pan W Wang J Zhan H Liu S . Targeting tumor immunosuppressive microenvironment for pancreatic cancer immunotherapy: Current research and future perspective. Front Oncol. (2023) 13:1166860. doi: 10.3389/fonc.2023.1166860 37064113 PMC10090519

[6] Nejman D Livyatan I Fuks G Gavert N Zwang Y Geller LT . The human tumor microbiome is composed of tumor type-specific intracellular bacteria. Science. (2020) 368:973–80. doi: 10.1126/science.aay9189 32467386 PMC7757858

[7] Narunsky-Haziza L Sepich-Poore GD Livyatan I Asraf O Martino C Nejman D . Pan-cancer analyses reveal cancer-type-specific fungal ecologies and bacteriome interactions. Cell. (2022) 185:3789–3806.e17. doi: 10.1016/j.cell.2022.09.005 36179670 PMC9567272

[8] Lin A Xiong M Jiang A Huang L Wong HZH Feng S . The microbiome in cancer. Imeta. (2025) 4:e70070. doi: 10.1002/imt2.70070 41112042 PMC12528007

[9] Geller LT Barzily-Rokni M Danino T Jonas OH Shental N Nejman D . Potential role of intratumor bacteria in mediating tumor resistance to the chemotherapeutic drug gemcitabine. Science. (2017) 357:1156–60. doi: 10.1126/science.aah5043 28912244 PMC5727343

[10] Guo W Zhang Y Guo S Mei Z Liao H Dong H . Tumor microbiome contributes to an aggressive phenotype in the basal-like subtype of pancreatic cancer. Commun Biol. (2021) 4:1019. doi: 10.1038/s42003-021-02557-5 34465850 PMC8408135

[11] Chakladar J Kuo SZ Castaneda G Li WT Gnanasekar A Yu MA . The pancreatic microbiome is associated with carcinogenesis and worse prognosis in males and smokers. Cancers (Basel). (2020) 12:2672. doi: 10.3390/cancers12092672 32962112 PMC7565819

[12] McAllister F Khan MAW Helmink B Wargo JA . The tumor microbiome in pancreatic cancer: Bacteria and beyond. Cancer Cell. (2019) 36:577–9. doi: 10.1016/j.ccell.2019.11.004 31951558

[13] Eisenhofer R Minich JJ Marotz C Cooper A Knight R Weyrich LS . Contamination in low microbial biomass microbiome studies: Issues and recommendations. Trends Microbiol. (2019) 27:105–17. doi: 10.1016/j.tim.2018.11.003 30497919

[14] Li Y Ma A Johnson E Eng C De S Jiang S . The new microbiome on the block: Challenges and opportunities of using human tumor sequencing data to study microbes. Nat Methods. (2025) 22:1788–99. doi: 10.1038/s41592-025-02807-y 40954302 PMC12701766

[15] Daniel N Farinella R Chatziioannou AC Jenab M Mayén AL Rizzato C . Genetically predicted gut bacteria, circulating bacteria-associated metabolites and pancreatic ductal adenocarcinoma: A Mendelian randomisation study. Sci Rep. (2024) 14:25144. doi: 10.1038/s41598-024-77431-5 39448785 PMC11502931

[16] Dohlman AB Arguijo Mendoza D Ding S Gao M Dressman H Iliev ID . The cancer microbiome atlas: A pan-cancer comparative analysis to distinguish tissue-resident microbiota from contaminants. Cell Host Microbe. (2021) 29:281–298.e5. doi: 10.1016/j.chom.2020.12.001 33382980 PMC7878430

[17] Aykut B Pushalkar S Chen R Li Q Abengozar R Kim JI . The fungal mycobiome promotes pancreatic oncogenesis via activation of MBL. Nature. (2019) 574:264–7. doi: 10.1038/s41586-019-1608-2 31578522 PMC6858566

[18] Bai D Fang O Li C Cai B Tan X Jiang M . Accu16S/AccuITS: Accurate and broadly applicable amplicon sequencing for absolute microbiome quantification. Imeta. (2026) 5:e70116. doi: 10.1002/imt2.70116 42099459 PMC13147958

[19] Pu Y Zhou X Cai H Lou T Liu C Kong M . Impact of DNA extraction methods on gut microbiome profiles: A comparative metagenomic study. Phenomics. (2025) 5:76–90. doi: 10.1007/s43657-025-00232-x 40313603 PMC12040788

[20] Wu H Forslund S Wang Z Zhao G . Human gut microbiome researches over the last decade: Current challenges and future directions. Phenomics. (2025) 5:1–7. doi: 10.1007/s43657-023-00131-z 40313604 PMC12040780

[21] Thomas RM Jobin C . Microbiota in pancreatic health and disease: The next frontier in microbiome research. Nat Rev Gastroenterol Hepatol. (2020) 17:53–64. doi: 10.1038/s41575-019-0242-7 31811279

[22] Riquelme E Zhang Y Zhang L Montiel M Zoltan M Dong W . Tumor microbiome diversity and composition influence pancreatic cancer outcomes. Cell. (2019) 178:795–806.e12. doi: 10.1016/j.cell.2019.07.008 31398337 PMC7288240

[23] Mitsuhashi K Nosho K Sukawa Y Matsunaga Y Ito M Kurihara H . Association of Fusobacterium species in pancreatic cancer tissues with molecular features and prognosis. Oncotarget. (2015) 6:7209–20. doi: 10.18632/oncotarget.3109 25797243 PMC4466679

[24] Awad D Attebury H Hong R Kim K Zhang L Bischoff A . Isolation of bacteria and fungi from human pancreatic tumors and duodenum. Gut Microbes. (2026) 18:2695522. doi: 10.1080/19490976.2026.2695522 42411381 PMC13348923

[25] Jayakrishnan T Sangwan N Nair KG Kamath SD Patel MH Joyce D . Tumor microbiome differences in early-onset versus average-onset pancreatic adenocarcinoma. ESMO Gastrointest Oncol. (2025) 9:100194. doi: 10.1016/j.esmogo.2025.100194 41647993 PMC12836659

[26] Fang Y Liu X Ren J Wang X Zhou F Huang S . Integrated analysis of microbiome and metabolome reveals signatures in PDAC tumorigenesis and prognosis. Microbiol Spectr. (2024) 12:e0096224. doi: 10.1128/spectrum.00962-24 39387592 PMC11540152

[27] Podgorny PM Weiss S Bang C Rühlemann M Wiese ML Völzke H . The fecal mycobiome in chronic pancreatitis is characterized by an increase in Candida species and Nakaseomyces. Clin Transl Gastroenterol. (2025) 16:e00855. doi: 10.14309/ctg.0000000000000855 40358410 PMC12330351

[28] Fletcher AA Kelly MS Eckhoff AM Allen PJ . Revisiting the intrinsic mycobiome in pancreatic cancer. Nature. (2023) 620:E1–6. doi: 10.1038/s41586-023-06292-1 37532819 PMC11062486

[29] Xu F Saxena D Pushalkar S Miller G . Reply to: Revisiting the intrinsic mycobiome in pancreatic cancer. Nature. (2023) 620:E7–9. doi: 10.1038/s41586-023-06293-0 37532815

[30] Peng JF Li M Niu T Li J Niu W Zheng M . Intratumoral Enterobacter hormaechei drives gemcitabine resistance in pancreatic cancer via cdd L-mediated drug inactivation. Imeta. (2026) 5:e70126. doi: 10.1002/imt2.70126 42099454 PMC13147963

[31] Pushalkar S Hundeyin M Daley D Zambirinis CP Kurz E Mishra A . The pancreatic cancer microbiome promotes oncogenesis by induction of innate and adaptive immune suppression. Cancer Discov. (2018) 8:403–16. doi: 10.1158/2159-8290.CD-17-1134 29567829 PMC6225783

[32] Xu M Guan S Zhong C Ma M Tao L Huang G . Characterizing the microbiome of “sterile” organs in experimental mice and evidence of translocation of bacteria from the gut to other internal organs. Imeta. (2025) 4:e70081. doi: 10.1002/imt2.70081 41112047 PMC12527995

[33] Guo X Shao Y . Role of the oral-gut microbiota axis in pancreatic cancer: A new perspective on tumor pathophysiology, diagnosis, and treatment. Mol Med. (2025) 31:103. doi: 10.1186/s10020-025-01166-w 40102723 PMC11917121

[34] Zhao L Peng S Ge M Xing B Zhao X Yang T . Gut-to-tumor translocation of multidrug-resistant Klebsiella pneumoniae shapes the microbiome and chemoresistance in pancreatic cancer. Front Cell Infect Microbiol. (2025) 15:1694479. doi: 10.3389/fcimb.2025.1694479 41409546 PMC12705542

[35] Michaud DS Izard J Wilhelm-Benartzi CS You DH Grote VA Tjønneland A . Plasma antibodies to oral bacteria and risk of pancreatic cancer in a large European prospective cohort study. Gut. (2013) 62:1764–70. doi: 10.1136/gutjnl-2012-303006 22990306 PMC3815505

[36] Meng Y Wu F Kwak S Wang C Usyk M Freedman ND . Oral bacterial and fungal microbiome and subsequent risk for pancreatic cancer. JAMA Oncol. (2025) 11:1331–40. doi: 10.1001/jamaoncol.2025.3377 40965868 PMC12447289

[37] Fan X Alekseyenko AV Wu J Peters BA Jacobs EJ Gapstur SM . Human oral microbiome and prospective risk for pancreatic cancer: A population-based nested case-control study. Gut. (2018) 67:120–7. doi: 10.1136/gutjnl-2016-312580 27742762 PMC5607064

[38] Kohi S Macgregor-Das A Dbouk M Yoshida T Chuidian M Abe T . Alterations in the duodenal fluid microbiome of patients with pancreatic cancer. Clin Gastroenterol Hepatol. (2022) 20:e196–227. doi: 10.1016/j.cgh.2020.11.006 33161160 PMC8120597

[39] Half E Keren N Reshef L Dorfman T Lachter I Kluger Y . Fecal microbiome signatures of pancreatic cancer patients. Sci Rep. (2019) 9:16801. doi: 10.1038/s41598-019-53041-4 31727922 PMC6856127

[40] Li P Zhang H Dai M . Current status and prospect of gut and oral microbiome in pancreatic cancer: Clinical and translational perspectives. Cancer Lett. (2024) 604:217274. doi: 10.1016/j.canlet.2024.217274 39307411

[41] Qi Z Qi P Jiang X Qi P . Progress in intestinal homeostasis and mechanisms of pancreatic cancer. Phenomics. (2025) 5:446–56. doi: 10.1007/s43657-024-00189-3 41001434 PMC12457279

[42] Galeano Niño JL Wu H LaCourse KD Kempchinsky AG Baryiames A Barber B . Effect of the intratumoral microbiota on spatial and cellular heterogeneity in cancer. Nature. (2022) 611:810–7. doi: 10.1038/s41586-022-05435-0 36385528 PMC9684076

[43] Moncada R Barkley D Wagner F Chiodin M Devlin JC Baron M . Integrating microarray-based spatial transcriptomics and single-cell RNA-seq reveals tissue architecture in pancreatic ductal adenocarcinomas. Nat Biotechnol. (2020) 38:333–42. doi: 10.1038/s41587-019-0392-8 31932730

[44] Gaiser RA Halimi A Alkharaan H Lu L Davanian H Healy K . Enrichment of oral microbiota in early cystic precursors to invasive pancreatic cancer. Gut. (2019) 68:2186–94. doi: 10.1136/gutjnl-2018-317458 30872392 PMC6872446

[45] Panebianco C Villani A Pisati F Orsenigo F Ulaszewska M Latiano TP . Butyrate, a postbiotic of intestinal bacteria, affects pancreatic cancer and gemcitabine response in *in vitro* and *in vivo* models. BioMed Pharmacother. (2022) 151:113163. doi: 10.1016/j.biopha.2022.113163 35617803

[46] Ferreira TM Leonel AJ Melo MA Santos RRG Cara DC Cardoso VN . Oral supplementation of butyrate reduces mucositis and intestinal permeability associated with 5-Fluorouracil administration. Lipids. (2012) 47:669–78. doi: 10.1007/s11745-012-3680-3 22648862

[47] Sulekha Suresh D Jain T Dudeja V Iyer S Dudeja V . From microbiome to malignancy: Unveiling the gut microbiome dynamics in pancreatic carcinogenesis. Int J Mol Sci. (2025) 26:3112. doi: 10.3390/ijms26073112 40243755 PMC11988718

[48] Zhou X Chen X Davis MM Snyder MP . Embracing interpersonal variability of microbiome in precision medicine. Phenomics. (2025) 5:8–13. doi: 10.1007/s43657-024-00201-w 40313605 PMC12040794

[49] Ge Y Lu J Puiu D Revsine M Salzberg SL . Comprehensive analysis of microbial content in whole-genome sequencing samples from The Cancer Genome Atlas project. Sci Transl Med. (2025) 17:eads6335. doi: 10.1126/scitranslmed.ads6335 40901923 PMC12821378

[50] Pleguezuelos-Manzano C Puschhof J Rosendahl Huber A van Hoeck A Wood HM Nomburg J . Mutational signature in colorectal cancer caused by genotoxic pks+ E. coli. Nature. (2020) 580:269–73. doi: 10.1038/s41586-020-2080-8 32106218 PMC8142898

[51] Kang SS Sim JR Yun CH Han SH . Lipoteichoic acids as a major virulence factor causing inflammatory responses via Toll-like receptor 2. Arch Pharm Res. (2016) 39:1519–29. doi: 10.1007/s12272-016-0804-y 27498542

[52] Wolf AJ Underhill DM . Peptidoglycan recognition by the innate immune system. Nat Rev Immunol. (2018) 18:243–54. doi: 10.1038/nri.2017.136 29292393

[53] Sethi V Kurtom S Tarique M Lavania S Malchiodi Z Hellmund L . Gut microbiota promotes tumor growth in mice by modulating immune response. Gastroenterology. (2018) 155:33–37.e6. doi: 10.1053/j.gastro.2018.04.001 29630898 PMC6035070

[54] Karin M Lawrence T Nizet V . Innate immunity gone awry: linking microbial infections to chronic inflammation and cancer. Cell. (2006) 124:823–35. doi: 10.1016/j.cell.2006.02.016 16497591

[55] Ochi A Nguyen AH Bedrosian AS Mushlin HM Zarbakhsh S Barilla R . MyD88 inhibition amplifies dendritic cell capacity to promote pancreatic carcinogenesis via Th2 cells. J Exp Med. (2012) 209:1671–87. doi: 10.1084/jem.20111706 22908323 PMC3428946

[56] Buscail L Bournet B Cordelier P . Role of oncogenic KRAS in the diagnosis, prognosis and treatment of pancreatic cancer. Nat Rev Gastroenterol Hepatol. (2020) 17:153–68. doi: 10.1038/s41575-019-0245-4 32005945

[57] Gurreri E Genovese G Perelli L Agostini A Piro G Carbone C . KRAS-dependency in pancreatic ductal adenocarcinoma: mechanisms of escaping in resistance to KRAS inhibitors and perspectives of therapy. Int J Mol Sci. (2023) 24:9313. doi: 10.3390/ijms24119313 37298264 PMC10253344

[58] Ma J Fu S Tan J Han Y Chen Y Deng X . Mechanistic foundations of KRAS-driven tumor ecosystems: integrating crosstalk among immune, metabolic, microbial, and stromal microenvironment. Adv Sci (Weinh). (2025) 12:e02714. doi: 10.1002/advs.202502714 40485603 PMC12376694

[59] Abe S Masuda A Matsumoto T Inoue J Toyama H Sakai A . Impact of intratumoral microbiome on tumor immunity and prognosis in human pancreatic ductal adenocarcinoma. J Gastroenterol. (2024) 59:250–62. doi: 10.1007/s00535-023-02069-5 38242997 PMC10904450

[60] D’Antonio DL Zenoniani A Umme S Piattelli A Curia MC . Intratumoral Fusobacterium nucleatum in pancreatic cancer: current and future perspectives. Pathogens. (2024) 14:2. doi: 10.3390/pathogens14010002 39860963 PMC11768203

[61] Ye C Liu X Liu Z Pan C Zhang X Zhao Z . Fusobacterium nucleatum in tumors: from tumorigenesis to tumor metastasis and tumor resistance. Cancer Biol Ther. (2024) 25:2306676. doi: 10.1080/15384047.2024.2306676 38289287 PMC10829845

[62] Alam A Levanduski E Denz P Villavicencio HS Bhatta M Alhorebi L . Fungal mycobiome drives IL-33 secretion and type 2 immunity in pancreatic cancer. Cancer Cell. (2022) 40:153–167.e11. doi: 10.1016/j.ccell.2022.01.003 35120601 PMC8847236

[63] Qian J Zhang X Wei B Tang Z Zhang B . The correlation between gut and intra-tumor microbiota and PDAC: etiology, diagnostics and therapeutics. Biochim Biophys Acta Rev Cancer. (2023) 1878:188943. doi: 10.1016/j.bbcan.2023.188943 37355177

[64] Guglietta S Li X Saxena D . Role of fungi in tumorigenesis: promises and challenges. Annu Rev Pathol. (2025) 20:459–82. doi: 10.1146/annurev-pathmechdis-111523-023524 39854185 PMC12277027

[65] Alves R Van Genechten W Van Dijck P . Tracking fungal pathogens on cancer: oncomicrobes or opportunistic bystanders? PloS Pathog. (2026) 22:e1014179. doi: 10.1371/journal.ppat.1014179 42096425 PMC13152125

[66] Estrin C Bertout S Bellet V . The hidden players: the mycobiome of pancreatic ductal adenocarcinoma tumors. Microbiol Res. (2026) 303:128392. doi: 10.1016/j.micres.2025.128392 41205302

[67] Alam A Mayengbam SS Senchanthisai S Bektas EI Dey P . Microbiome: a driver of pancreatic inflammation and tumorigenesis. Trends Cancer. (2026) 12:877–88. doi: 10.1016/j.trecan.2026.04.006 42128724 PMC13178113

[68] Mirzaei R Afaghi A Babakhani S Sohrabi MR Hosseini-Fard SR Babolhavaeji K . Role of microbiota-derived short-chain fatty acids in cancer development and prevention. BioMed Pharmacother. (2021) 139:111619. doi: 10.1016/j.biopha.2021.111619 33906079

[69] Davie JR . Inhibition of histone deacetylase activity by butyrate. J Nutr. (2003) 133:2485S–93S. doi: 10.1093/jn/133.7.2485S 12840228

[70] Singh N Gurav A Sivaprakasam S Brady E Padia R Shi H . Activation of Gpr109a, receptor for niacin and the commensal metabolite butyrate, suppresses colonic inflammation and carcinogenesis. Immunity. (2014) 40:128–39. doi: 10.1016/j.immuni.2013.12.007 24412617 PMC4305274

[71] Abdelhalim KA . Short-chain fatty acids (SCFAs) from gastrointestinal disorders, metabolism, epigenetics, central nervous system to cancer - a mini-review. Chem Biol Interact. (2024) 388:110851. doi: 10.1016/j.cbi.2023.110851 38145797

[72] Bachem A Makhlouf C Binger KJ de Souza DP Tull D Hochheiser K . Microbiota-derived short-chain fatty acids promote the memory potential of antigen-activated CD8+ T cells. Immunity. (2019) 51:285–297.e5. doi: 10.1016/j.immuni.2019.06.002 31272808

[73] Kim MH Kang SG Park JH Yanagisawa M Kim CH . Short-chain fatty acids activate GPR41 and GPR43 on intestinal epithelial cells to promote inflammatory responses in mice. Gastroenterology. (2013) 145:396–406.e1-10. doi: 10.1053/j.gastro.2013.04.056 23665276

[74] Smith PM Howitt MR Panikov N Michaud M Gallini CA Bohlooly-Y M . The microbial metabolites, short-chain fatty acids, regulate colonic Treg cell homeostasis. Science. (2013) 341:569–73. doi: 10.1126/science.1241165 23828891 PMC3807819

[75] Ulven T . Short-chain free fatty acid receptors FFA2/GPR43 and FFA3/GPR41 as new potential therapeutic targets. Front Endocrinol (Lausanne). (2012) 3:111. doi: 10.3389/fendo.2012.00111 23060857 PMC3462324

[76] Zhong Y Zhang D Ding F Zhang H Guo Y Huang Q . Intratumoral microbiota-driven macrophage reprogramming in pancreatic cancer via Blautia metabolite 6-hydroxyhexanoic acid. Int Immunopharmacol. (2025) 164:115421. doi: 10.1016/j.intimp.2025.115421 40865404

[77] Murthy D Attri KS Shukla SK Thakur R Chaika NV He C . Cancer-associated fibroblast-derived acetate promotes pancreatic cancer development by altering polyamine metabolism via the ACSS2-SP1-SAT1 axis. Nat Cell Biol. (2024) 26:613–27. doi: 10.1038/s41556-024-01372-4 38429478 PMC11021164

[78] Cannon A Thompson C Hall BR Jain M Kumar S Batra SK . Desmoplasia in pancreatic ductal adenocarcinoma: insight into pathological function and therapeutic potential. Genes Cancer. (2018) 9:78–86. doi: 10.18632/genesandcancer.171 30108679 PMC6086006

[79] Biffi G Tuveson DA . Diversity and biology of cancer-associated fibroblasts. Physiol Rev. (2021) 101:147–76. doi: 10.1152/physrev.00048.2019 32466724 PMC7864232

[80] Omary MB Lugea A Lowe AW Pandol SJ . The pancreatic stellate cell: a star on the rise in pancreatic diseases. J Clin Invest. (2007) 117:50–9. doi: 10.1172/JCI30082 17200706 PMC1716214

[81] Hingorani SR Wang L Multani AS Combs C Deramaudt TB Hruban RH . Trp53R172H and KrasG12D cooperate to promote chromosomal instability and widely metastatic pancreatic ductal adenocarcinoma in mice. Cancer Cell. (2005) 7:469–83. doi: 10.1016/j.ccr.2005.04.023 15894267

[82] Udayasuryan B Ahmad RN Nguyen TTD Umaña A Monét Roberts L Sobol P . Fusobacterium nucleatum induces proliferation and migration in pancreatic cancer cells through host autocrine and paracrine signaling. Sci Signal. (2022) 15:eabn4948. doi: 10.1126/scisignal.abn4948 36256708 PMC9732933

[83] Neoptolemos JP Jones RP Büchler MW . Recurrence after resection of pancreatic ductal adenocarcinoma-reply. JAMA Surg. (2020) 155:362–3. doi: 10.1001/jamasurg.2019.5465 31895438

[84] Cruz MS Tintelnot J Gagliani N . Roles of microbiota in pancreatic cancer development and treatment. Gut Microbes. (2024) 16:2320280. doi: 10.1080/19490976.2024.2320280 38411395 PMC10900280

[85] Drost J Clevers H . Organoids in cancer research. Nat Rev Cancer. (2018) 18:407–18. doi: 10.1038/s41568-018-0007-6 29692415

[86] Zhang Z Wang T Xu M Zhang Z Wang H Xue J . Deciphering the pancreatic cancer microbiome in Mainland China: impact of Exiguobacterium/Bacillus ratio on tumor progression and prognostic significance. Pharmacol Res. (2024) 204:107197. doi: 10.1016/j.phrs.2024.107197 38692467

[87] Leng J Xu H Liu X Yang Y Ning C Sun L . Intratumoral microbiota of pancreatic ductal adenocarcinoma impact patient prognosis by influencing tumor microenvironment. Discov Oncol. (2024) 15:443. doi: 10.1007/s12672-024-01320-6 39271584 PMC11399525

[88] Li Y Chang RB Stone ML Delman D Markowitz K Xue Y . Multimodal immune phenotyping reveals microbial-T cell interactions that shape pancreatic cancer. Cell Rep Med. (2024) 5:101397. doi: 10.1016/j.xcrm.2024.101397 38307029 PMC10897543

[89] Wang W Qian C Wang T Jiang Y Zhou Y Liu K . A combination of faecal and intratumour microbial community profiling reveals novel diagnostic and prognostic biomarkers for pancreatic tumours. Clin Transl Med. (2024) 14:e1726. doi: 10.1002/ctm2.1726 38822473 PMC11142927

[90] Temel HY Kaymak Ö Kaplan S Bahcivanci B Gkoutos GV Acharjee A . Role of microbiota and microbiota-derived short-chain fatty acids in PDAC. Cancer Med. (2023) 12:5661–75. doi: 10.1002/cam4.5323 36205023 PMC10028056

[91] Fulop DJ Zylberberg HM Wu YL Aronson A Labiner AJ Wisnivesky J . Association of antibiotic receipt with survival among patients with metastatic pancreatic ductal adenocarcinoma receiving chemotherapy. JAMA Netw Open. (2023) 6:e234254. doi: 10.1001/jamanetworkopen.2023.4254 36951863 PMC10037151

[92] Kabwe M Dashper S Tucci J . The microbiome in pancreatic cancer-implications for diagnosis and precision bacteriophage therapy for this low survival disease. Front Cell Infect Microbiol. (2022) 12:871293. doi: 10.3389/fcimb.2022.871293 35663462 PMC9160434

[93] Pourali G Kazemi D Chadeganipour AS Arastonejad M Kashani SN Pourali R . Microbiome as a biomarker and therapeutic target in pancreatic cancer. BMC Microbiol. (2024) 24:16. doi: 10.1186/s12866-023-03166-4 38183010 PMC10768369

[94] Hu ZI Shia J Stadler ZK Varghese AM Capanu M Salo-Mullen E . Evaluating mismatch repair deficiency in pancreatic adenocarcinoma: challenges and recommendations. Clin Cancer Res. (2018) 24:1326–36. doi: 10.1158/1078-0432.CCR-17-3099 29367431 PMC5856632

[95] Zhu X Li K Liu G Wu R Zhang Y Wang S . Microbial metabolite butyrate promotes anti-PD-1 antitumor efficacy by modulating T cell receptor signaling of cytotoxic CD8 T cell. Gut Microbes. (2023) 15:2249143. doi: 10.1080/19490976.2023.2249143 37635362 PMC10464552

[96] Xiao W Chen Q Liu C Yu Y Liu T Jin Y . Gut microbiota in cancer: from molecular mechanisms to precision medicine applications. Imeta. (2025) 4:e70017. doi: 10.1002/imt2.70017 40236776 PMC11995170

[97] Mahadevia H Uson Junior PLS Wang J Borad M Babiker H . An overview of up-and-coming immune checkpoint inhibitors for pancreatic cancer. Expert Opin Pharmacother. (2024) 25:79–90. doi: 10.1080/14656566.2024.2304125 38193476

[98] González-Brito A Uribe-Herranz M . The potential role of short chain fatty acids improving ex vivo T and CAR-T cell fitness and expansion for cancer immunotherapies. Front Immunol. (2023) 14:1083303. doi: 10.3389/fimmu.2023.1083303 36742300 PMC9896517

[99] Shaji SG Patel P Mamani UF Guo Y Koirala S Lin CY . Delivery of a STING agonist using lipid nanoparticles inhibits pancreatic cancer growth. Int J Nanomedicine. (2024) 19:8769–78. doi: 10.2147/IJN.S462213 39220196 PMC11365503

[100] Korneenko TV Pestov NB Nevzorov IA Daks AA Trachuk KN Solopova ON . At the crossroads of the cGAS-cGAMP-STING pathway and the DNA damage response: implications for cancer progression and treatment. Pharm (Basel). (2023) 16:1675. doi: 10.3390/ph16121675 38139802 PMC10747911

[101] Meng J Cai H Sun Y Wen S Wu H Ren D . ITGA2 induces STING expression in pancreatic cancer by inducing DNMT1 degradation. Cell Oncol (Dordr). (2022) 45:1421–34. doi: 10.1007/s13402-022-00731-3 36331797 PMC12978104

[102] Chen Q Yin H He J Xie Y Wang W Xu H . Tumor microenvironment responsive CD8+ T cells and myeloid-derived suppressor cells to trigger CD73 inhibitor AB680-based synergistic therapy for pancreatic cancer. Adv Sci (Weinh). (2023) 10:e2302498. doi: 10.1002/advs.202302498 37867243 PMC10667825

[103] Van Laethem JL Borbath I Prenen H Geboes KP Lambert A Mitry E . Combining CD40 agonist mitazalimab with mFOLFIRINOX in previously untreated metastatic pancreatic ductal adenocarcinoma (OPTIMIZE-1): a single-arm, multicentre phase 1b/2 study. Lancet Oncol. (2024) 25:853–64. doi: 10.1016/S1470-2045(24)00263-8 38834087

[104] Trivieri N Panebianco C Villani A Pracella R Latiano TP Perri F . High levels of prebiotic resistant starch in diet modulate a specific pattern of miRNAs expression profile associated to a better overall survival in pancreatic cancer. Biomolecules. (2020) 11:26. doi: 10.3390/biom11010026 33383727 PMC7824309

[105] Bultman SJ . The microbiome and its potential as a cancer preventive intervention. Semin Oncol. (2016) 43:97–106. doi: 10.1053/j.seminoncol.2015.09.001 26970128 PMC4789109

[106] Zhang X Liu Q Liao Q Zhao Y . Pancreatic cancer, gut microbiota, and therapeutic efficacy. J Cancer. (2020) 11:2749–58. doi: 10.7150/jca.37445 32226493 PMC7086274

[107] DeFilipp Z Bloom PP Torres Soto M Mansour MK Sater MRA Huntley MH . Drug-resistant E. coli bacteremia transmitted by fecal microbiota transplant. N Engl J Med. (2019) 381:2043–50. doi: 10.1056/NEJMoa1910437 31665575

[108] Han ZY Fu ZJ Wang YZ Zhang C Chen QW An JX . Probiotics functionalized with a gallium-polyphenol network modulate the intratumor microbiota and promote anti-tumor immune responses in pancreatic cancer. Nat Commun. (2024) 15:7096. doi: 10.1038/s41467-024-51534-z 39154092 PMC11330462

[109] Chaib M Hafeez BB Mandil H Daria D Pingili AK Kumari S . Reprogramming of pancreatic adenocarcinoma immunosurveillance by a microbial probiotic siderophore. Commun Biol. (2022) 5:1181. doi: 10.1038/s42003-022-04102-4 36333531 PMC9636404

